# Supplementary material for: High-resolution model of Arabidopsis Photosystem II reveals the structural consequences of digitonin-extraction
Source: Sci Rep. 2021 Jul 30;11:15534. doi: 10.1038/s41598-021-94914-x (PMC8324835; doi:10.1038/s41598-021-94914-x)
Supplement: Supplementary file 1 — Supplementary Information 1. [file 41598_2021_94914_MOESM1_ESM.docx]

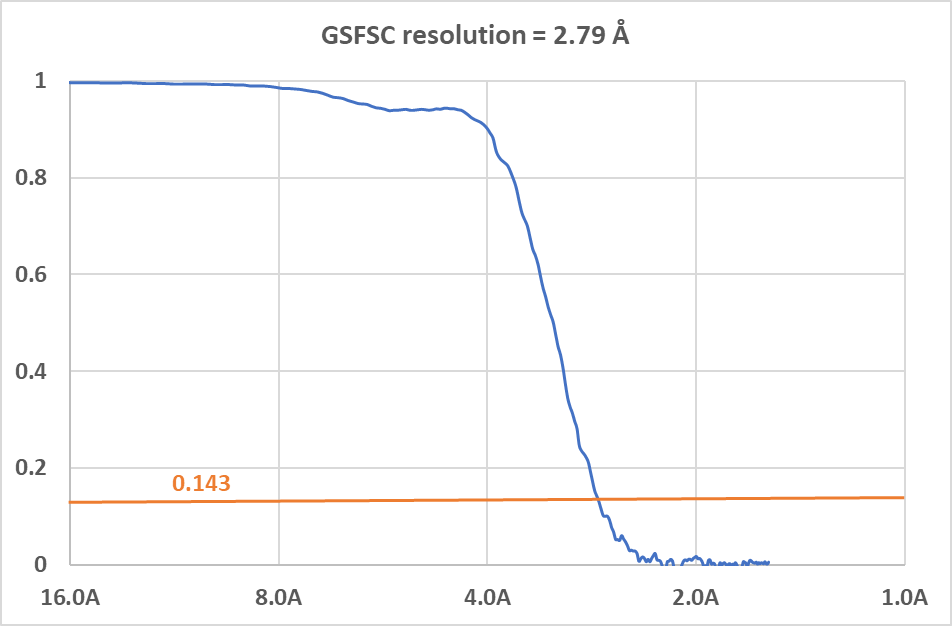


**A**


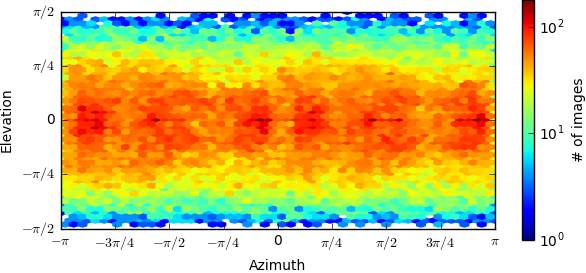


**B**

Figure S1 – A, Fourier Shell Correlation (FSC) curve of two independent refined datasets based on the golden-standard criterion for the C_2_S_2_ supercomplex. B, Plot for the angular distribution of particles that participated in the 3D reconstruction of the higher-resolution map.


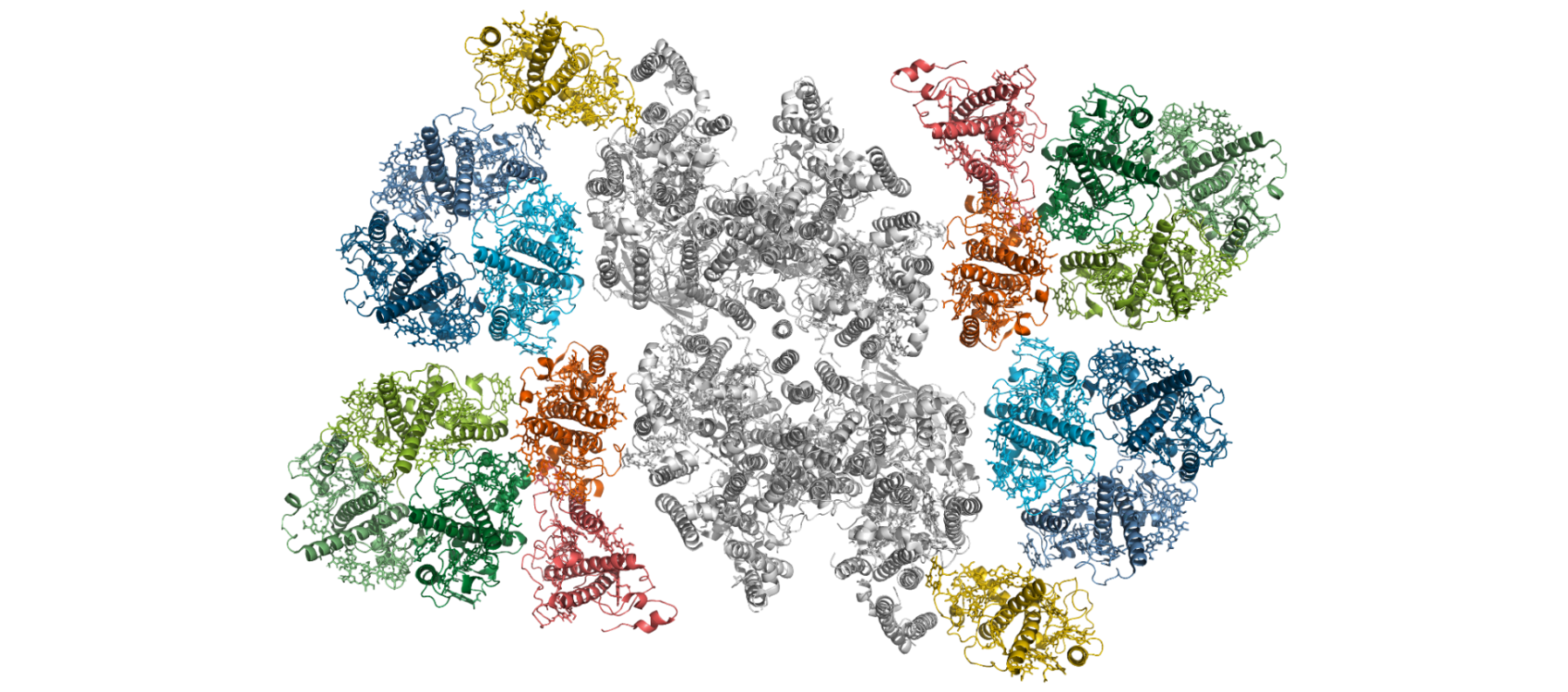

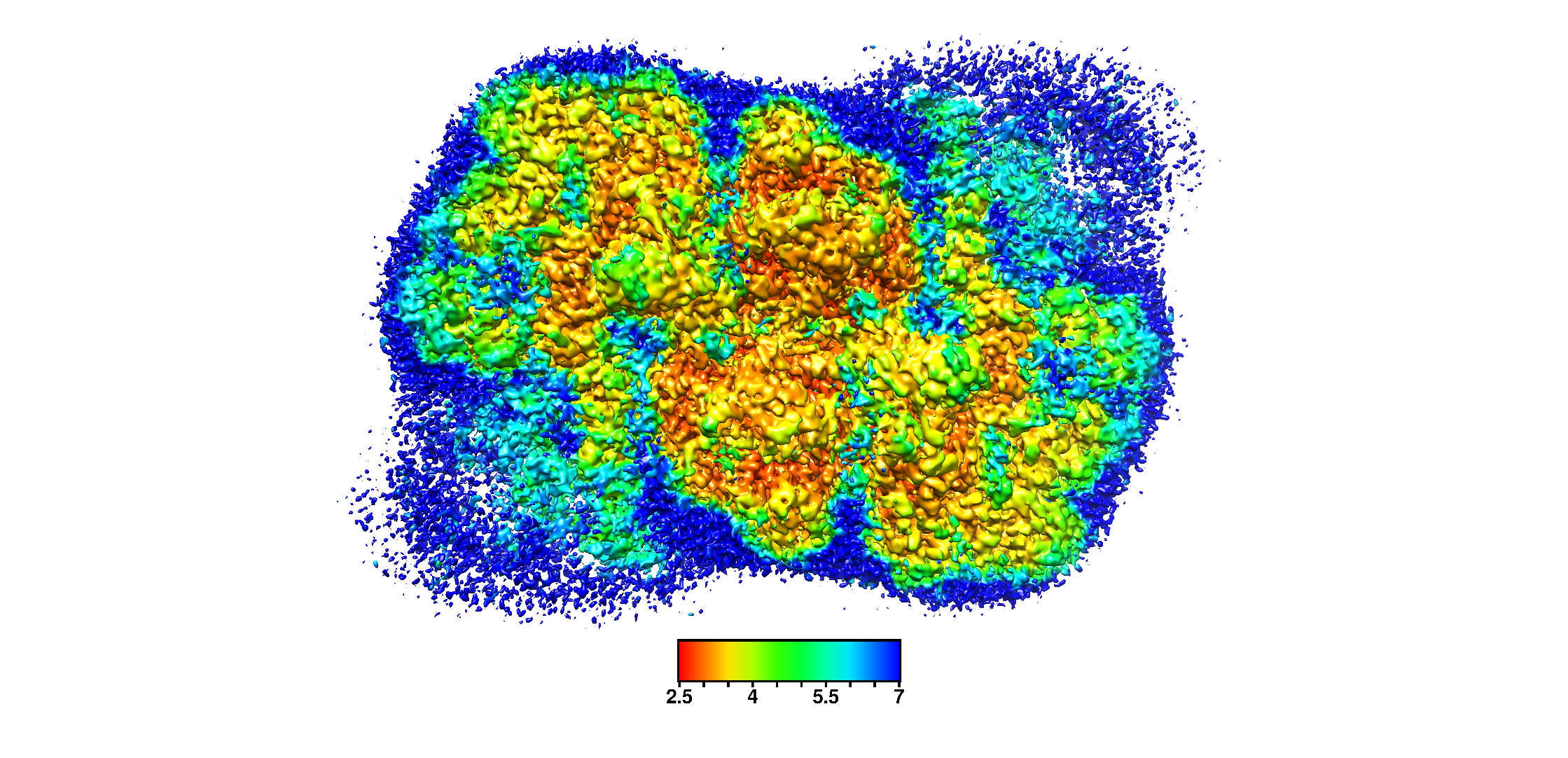

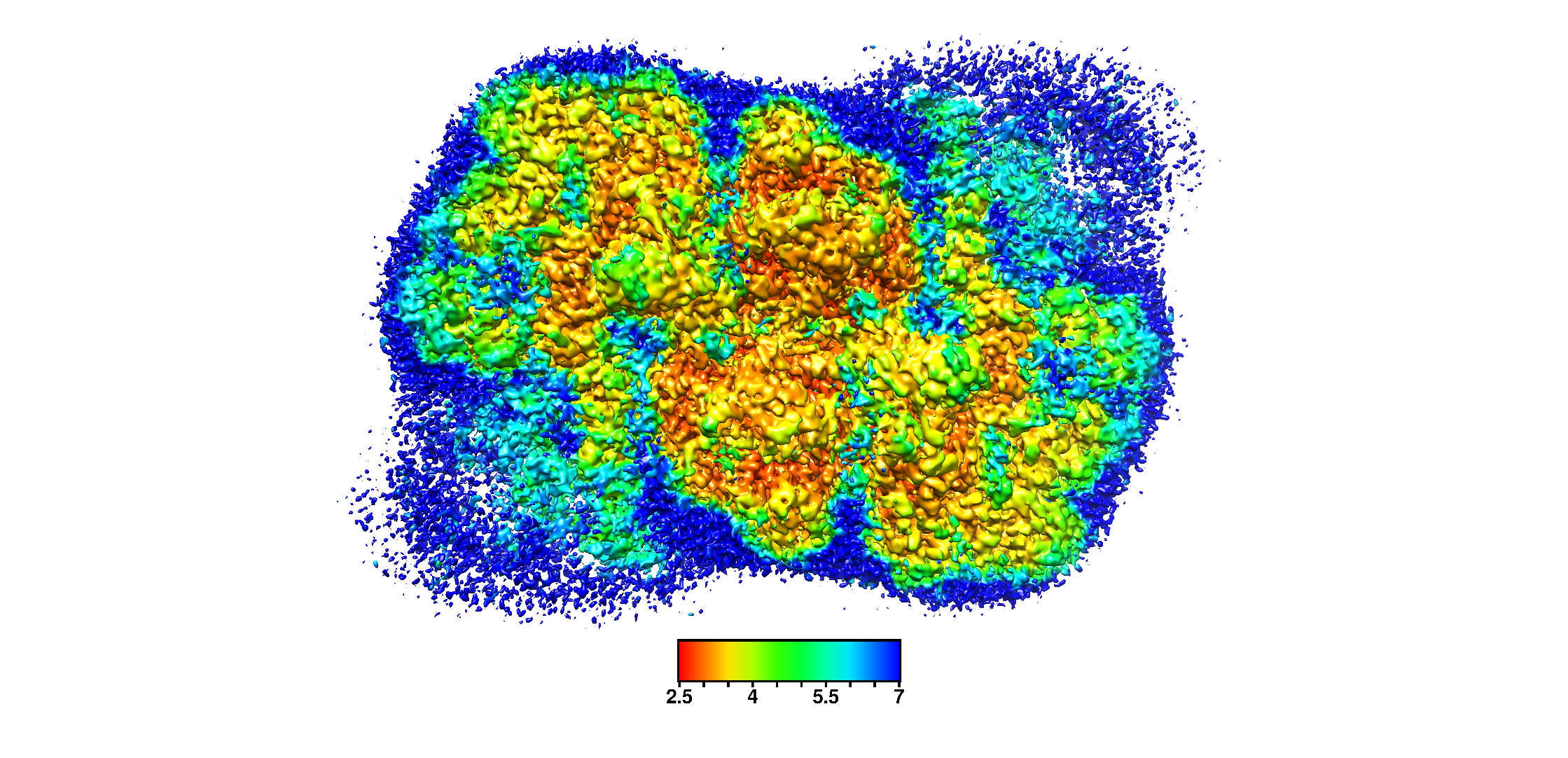


**Local Resolution (Å)**

Figure S2 – Top-view (stromal side) of our EM Map coloured according to local resolution. Local resolution was computed using cryoSPARC.

Figure S3 – Overview of the atomic model built from the single-particle Cryo-EM reconstruction of *Arabidopsis thaliana* Photosystem II C_2_S_2_M_2_ supercomplex, obtained from electron density map with overall resolution of 3.13 Å: A, top-view of the complex from the stromal side; B, side-view of the complex with the lumen side pointing down.


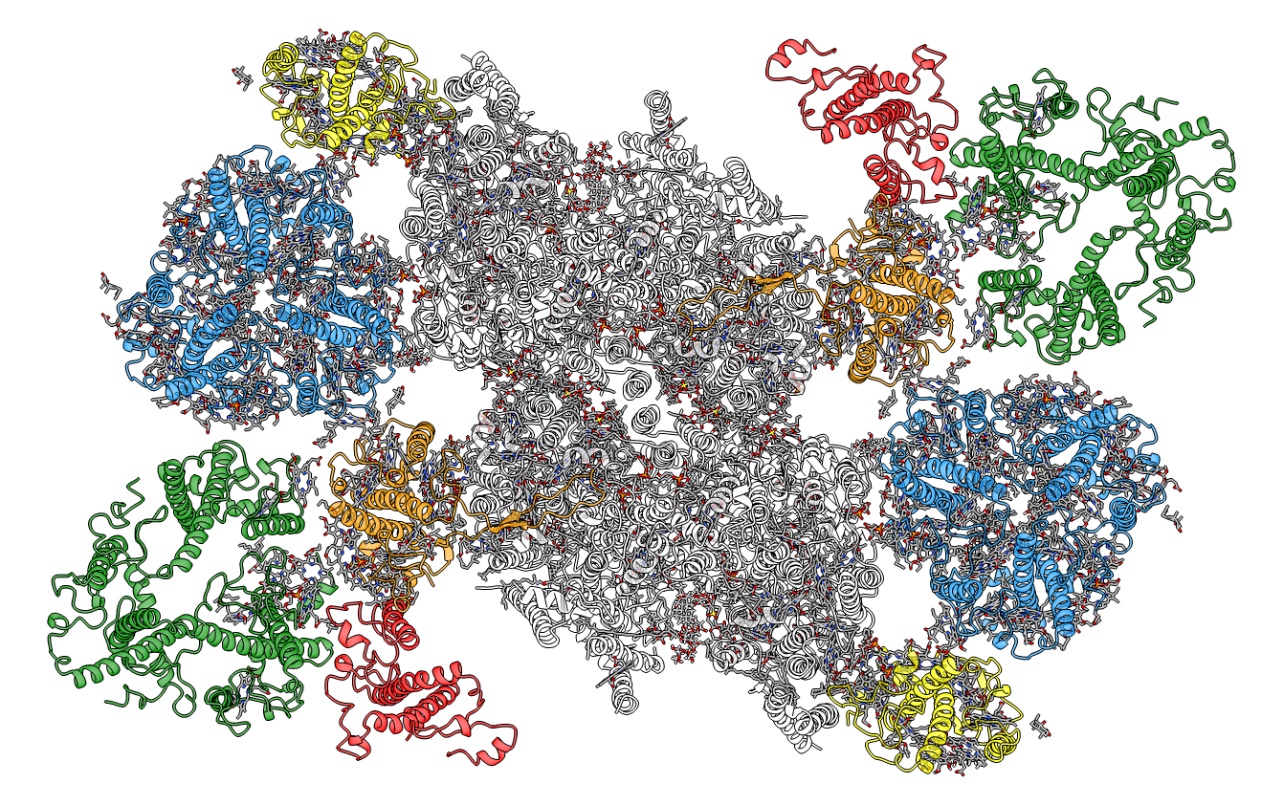

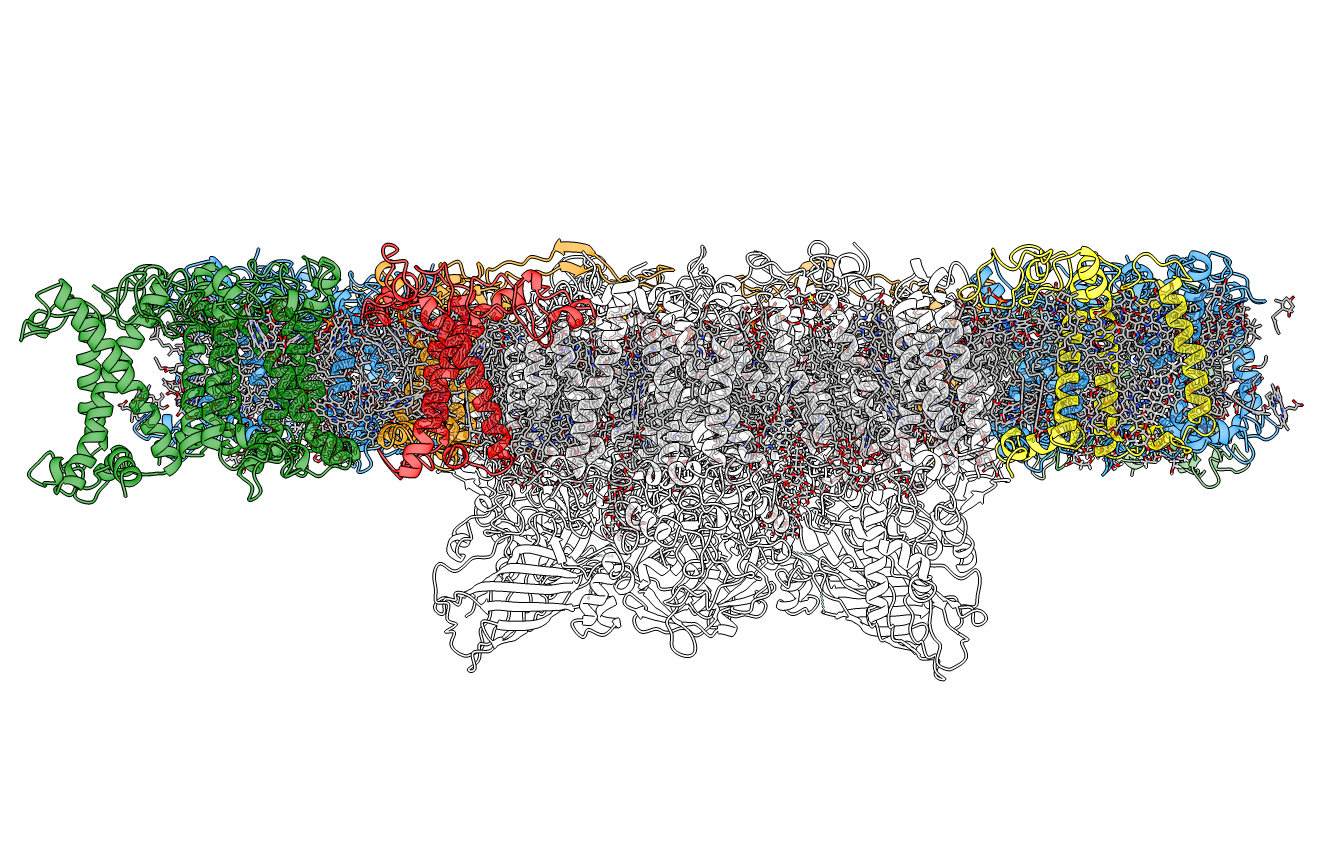


**A**

**180 Å**

**Core**

**S-LHCII**

**M-LHCII**

**CP24**

**CP26**

**CP29**

**B**

**280 Å**

**107 Å**

**50 Å**


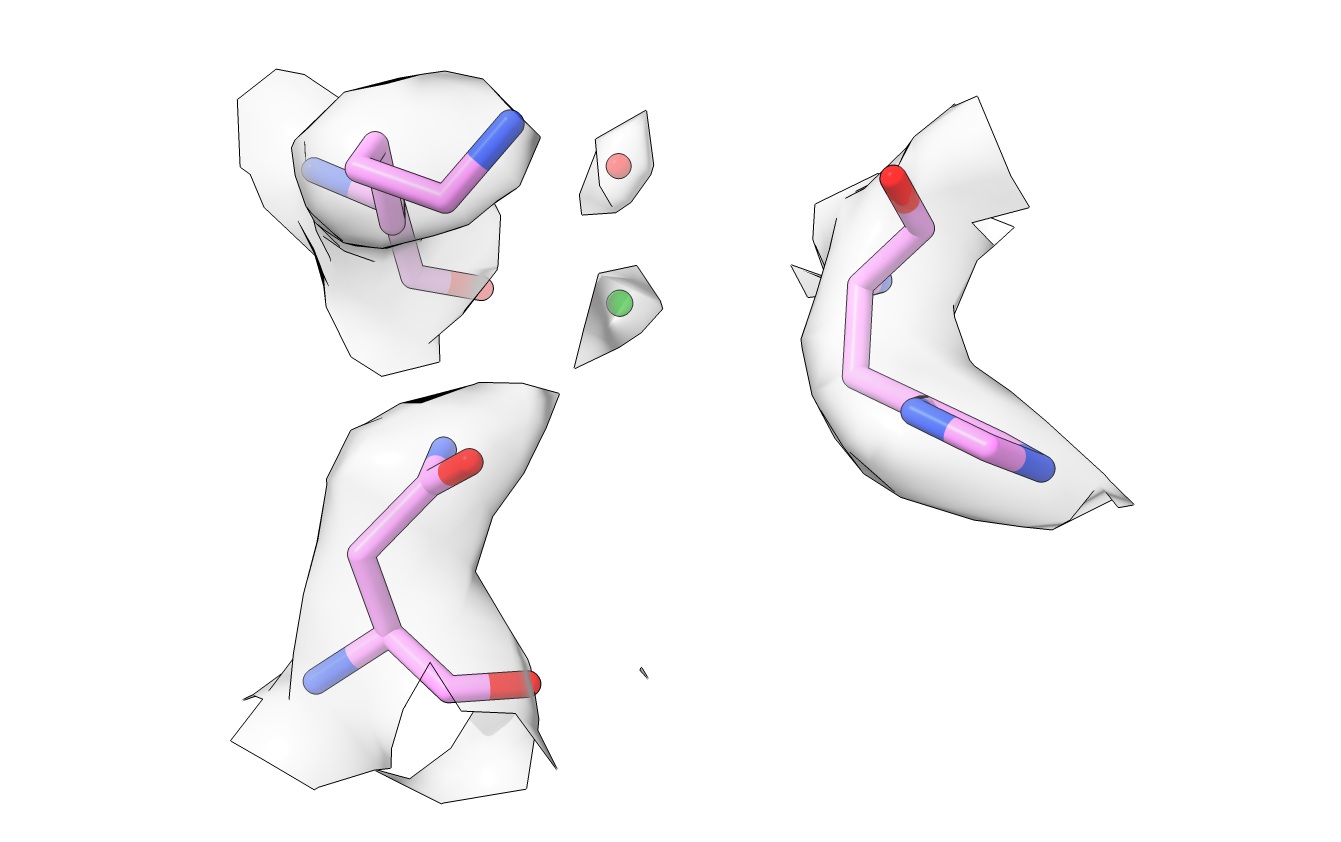


D1-ASN 181

D1-HIS 332

D2-LYS 318

H_2_O

Figure S4 - The binding site of the chloride anion (green) found in our EM map (surface coloured in grey).


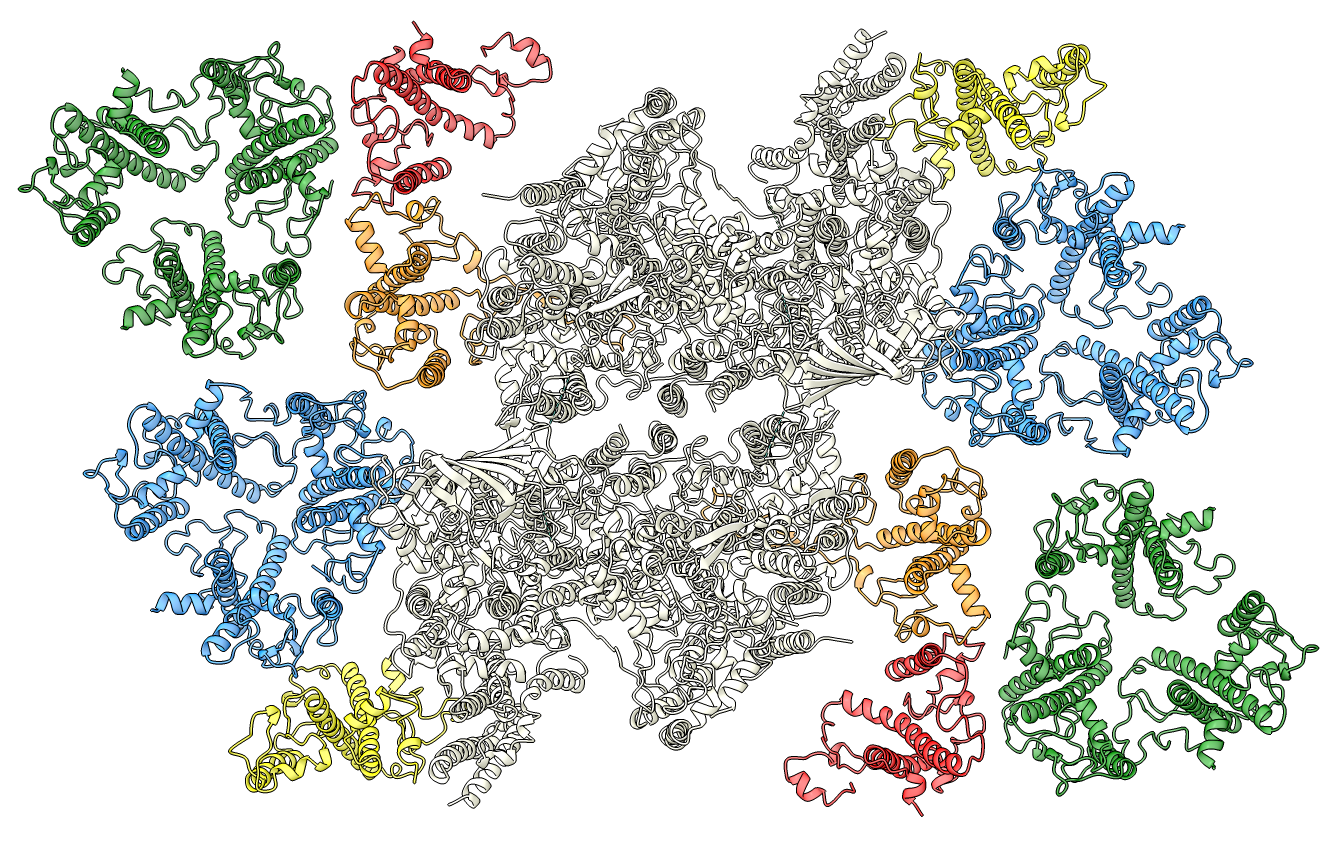

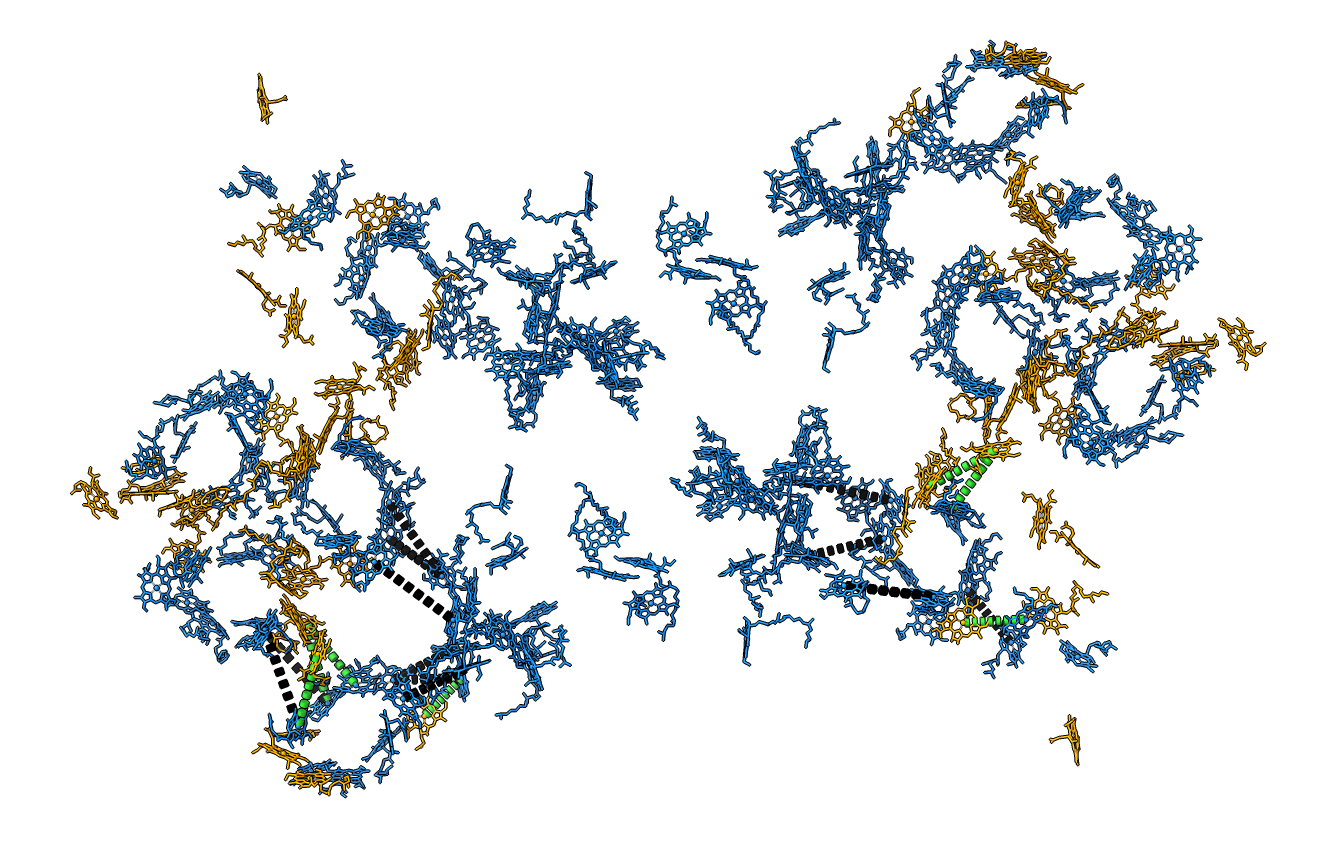


Figure S5 – Top-view of the distribution of chlorophylls in Arabidopsis PSII: chlorophyll *a* are coloured in blue and chlorophyll *b* in orange; the dashed lines represent the energy transfer between two couplings of chlorophyll *a* (black) and a coupling containing at least one chlorophyll *b* (green), corresponding distances can be seen in Table S6.


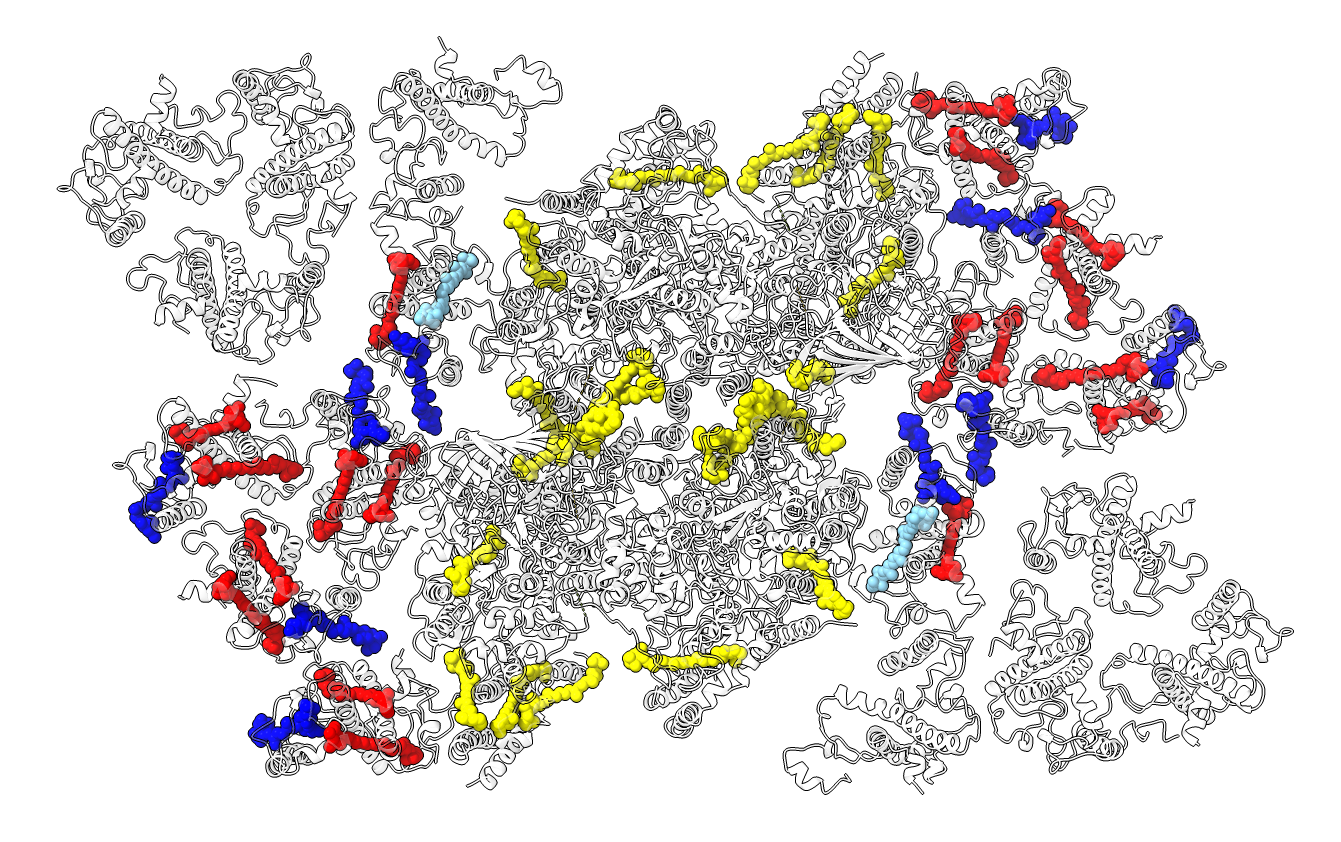
Figure S6 – Top-view of the distribution of carotenoid molecules in Arabidopsis PSII: beta-carotene (yellow), lutein (red), violaxhantin (light blue), neoxantin (blue).


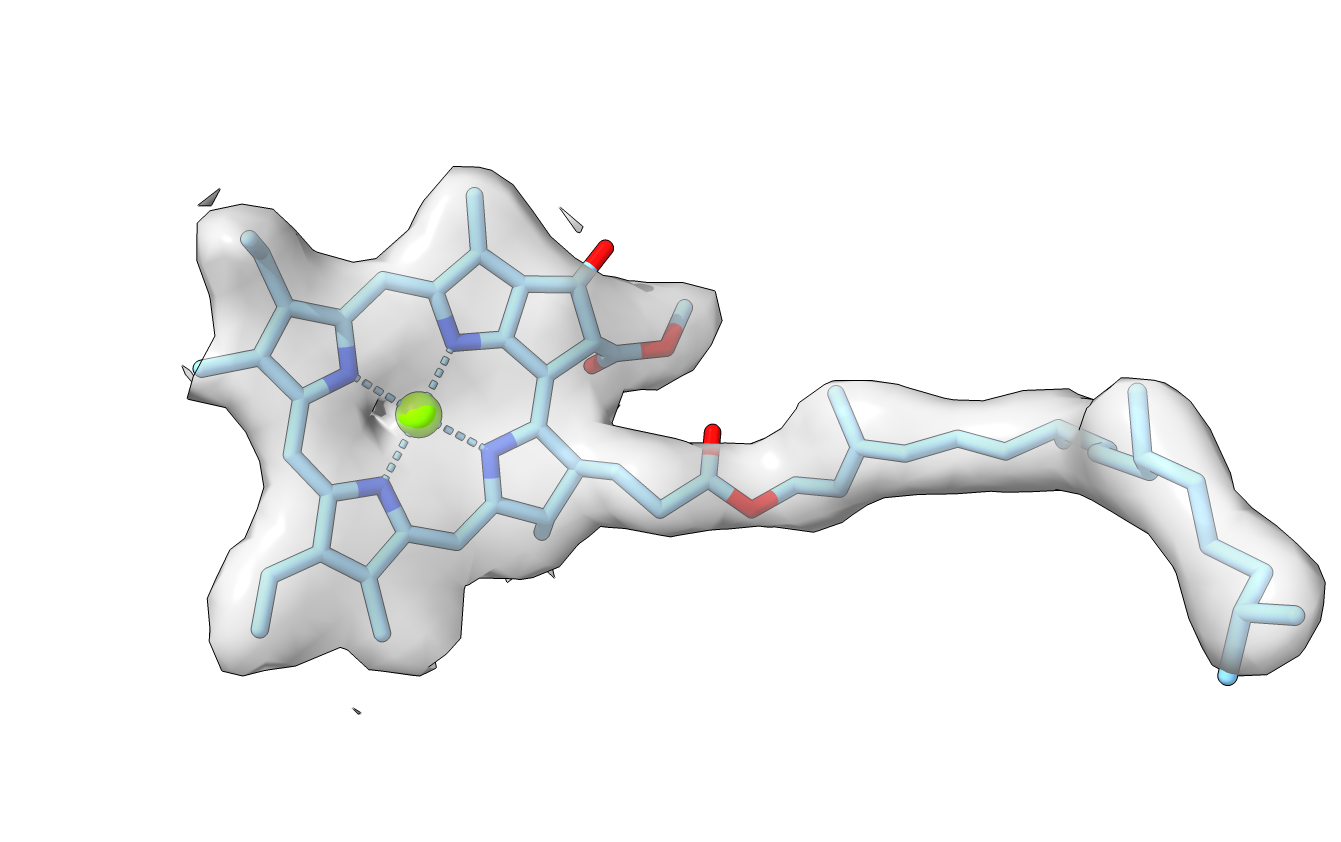


**CLA405**


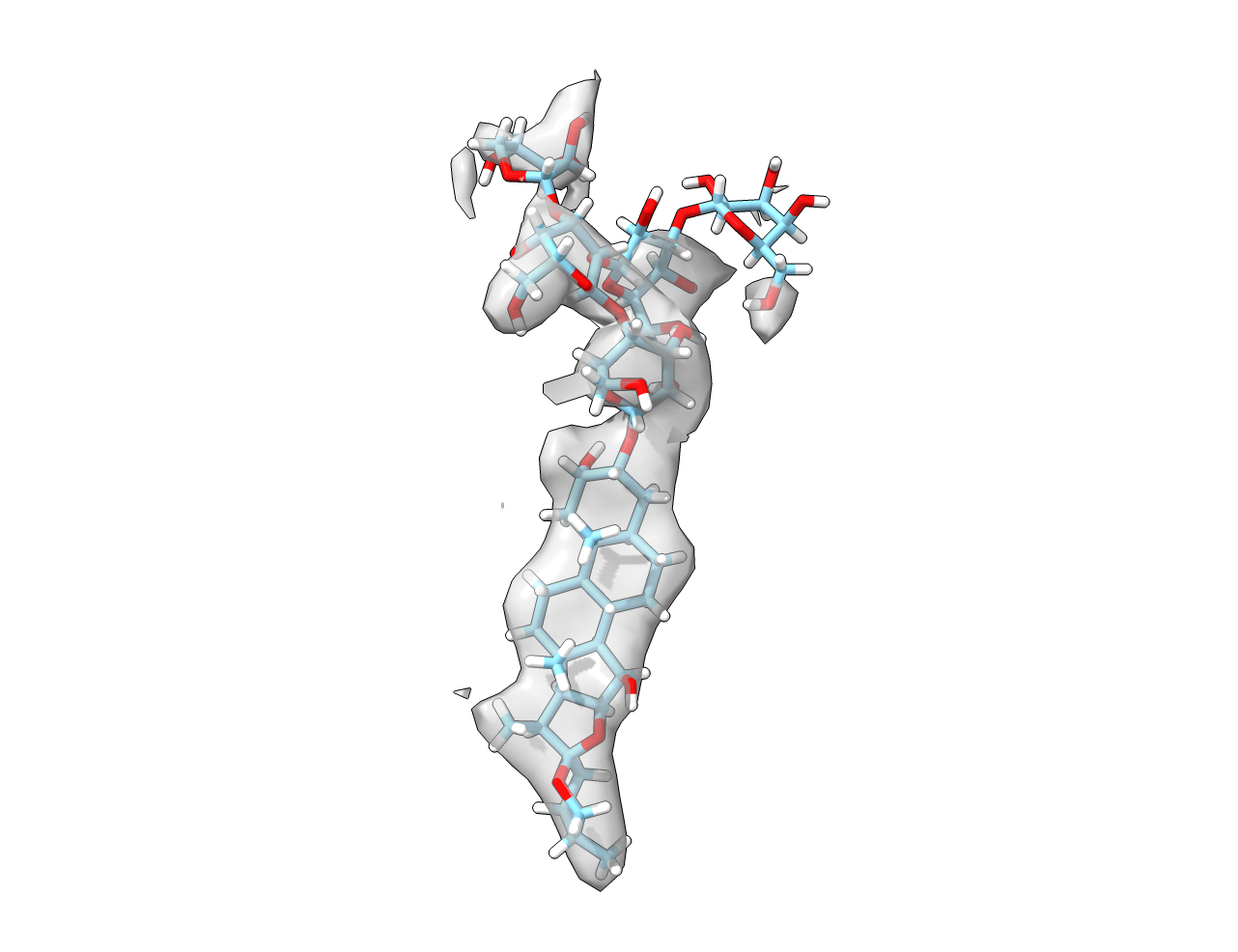


**AJP**

**PHO408**


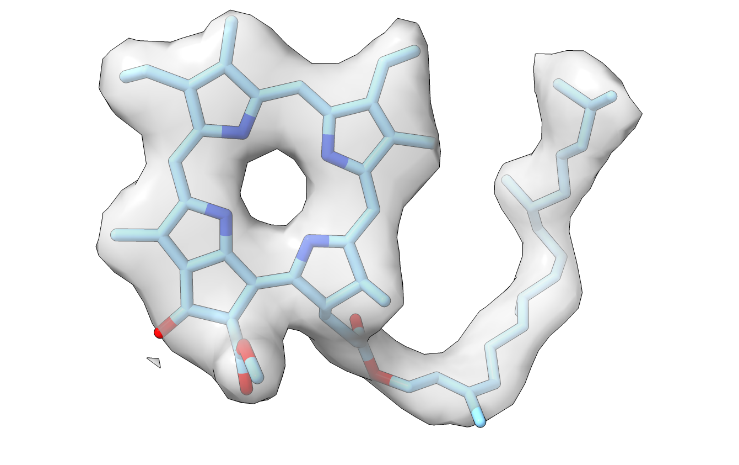


**BCR411**


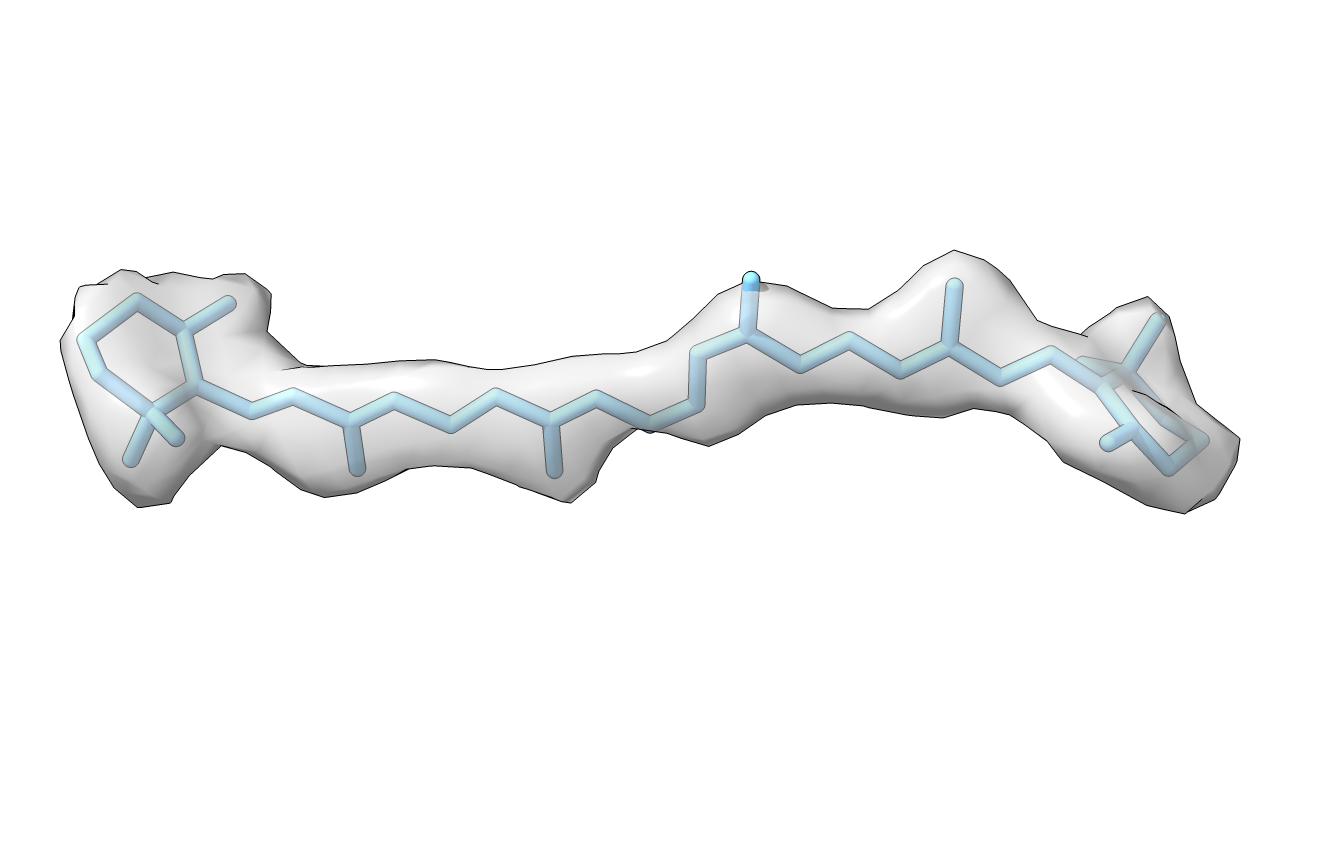


Figure S7 – Exemplars of some modelled ligands fitted into their respective densities: beta-carotene (BCR411), chlorophyll a (CLA405), pheophytin (PHO408) and a digitonin (AJP) molecule.


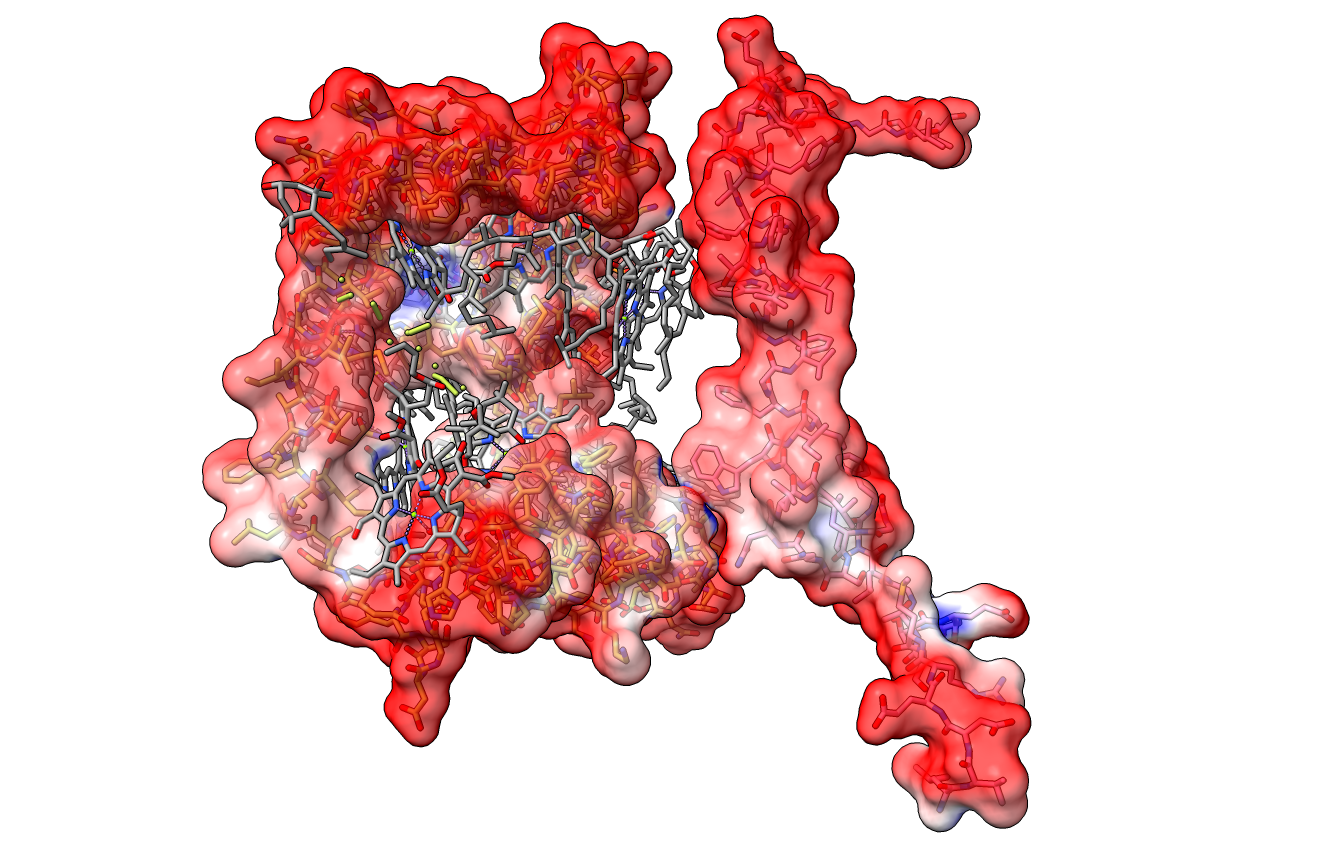

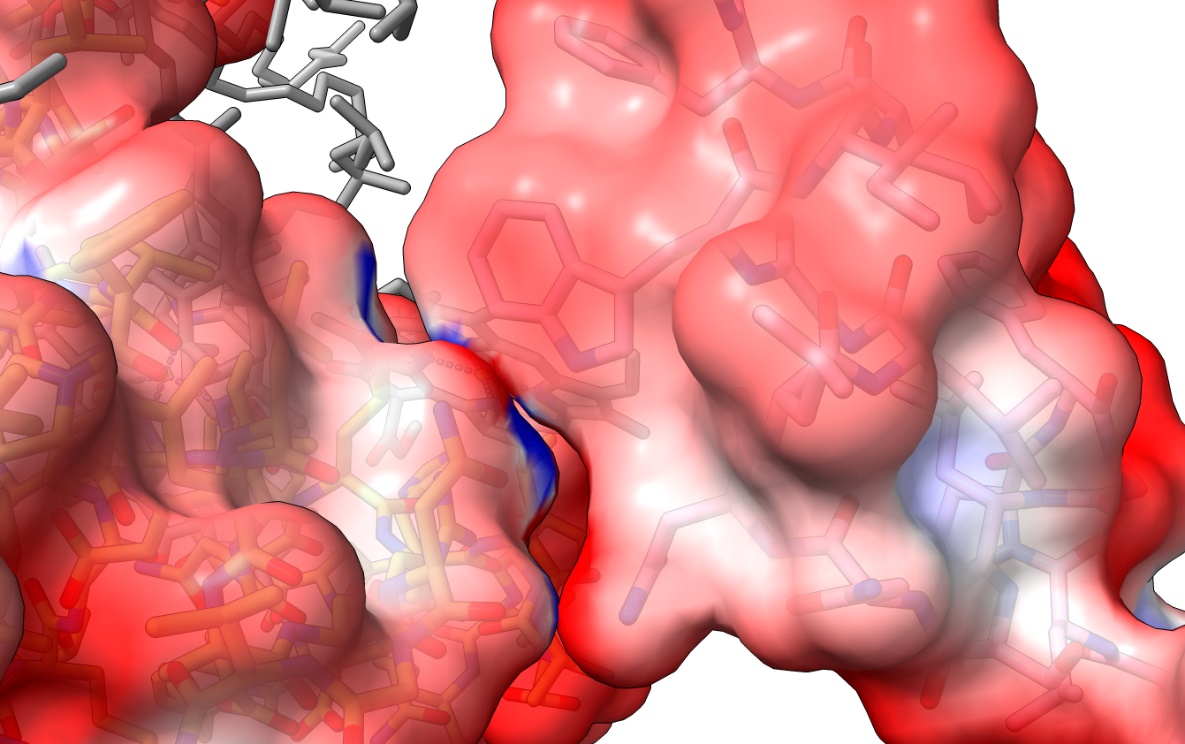


**TRP 103**

**ASN 122**

**ASN 99**

**PsbW**

**S-LHCII_A_**


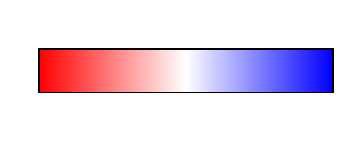


**- 3 kT/e**

**3 kT/e**

**Electrostatic potential**

Figure S8 - Electrostatic interactions between PsbW and monomer A of S-LHCII. The atomic model of PsbW and monomer A of S-LHCII, as well as the corresponding surface electrostatics of the polypeptide chains (slightly transparent to aid interpretation) are displayed.


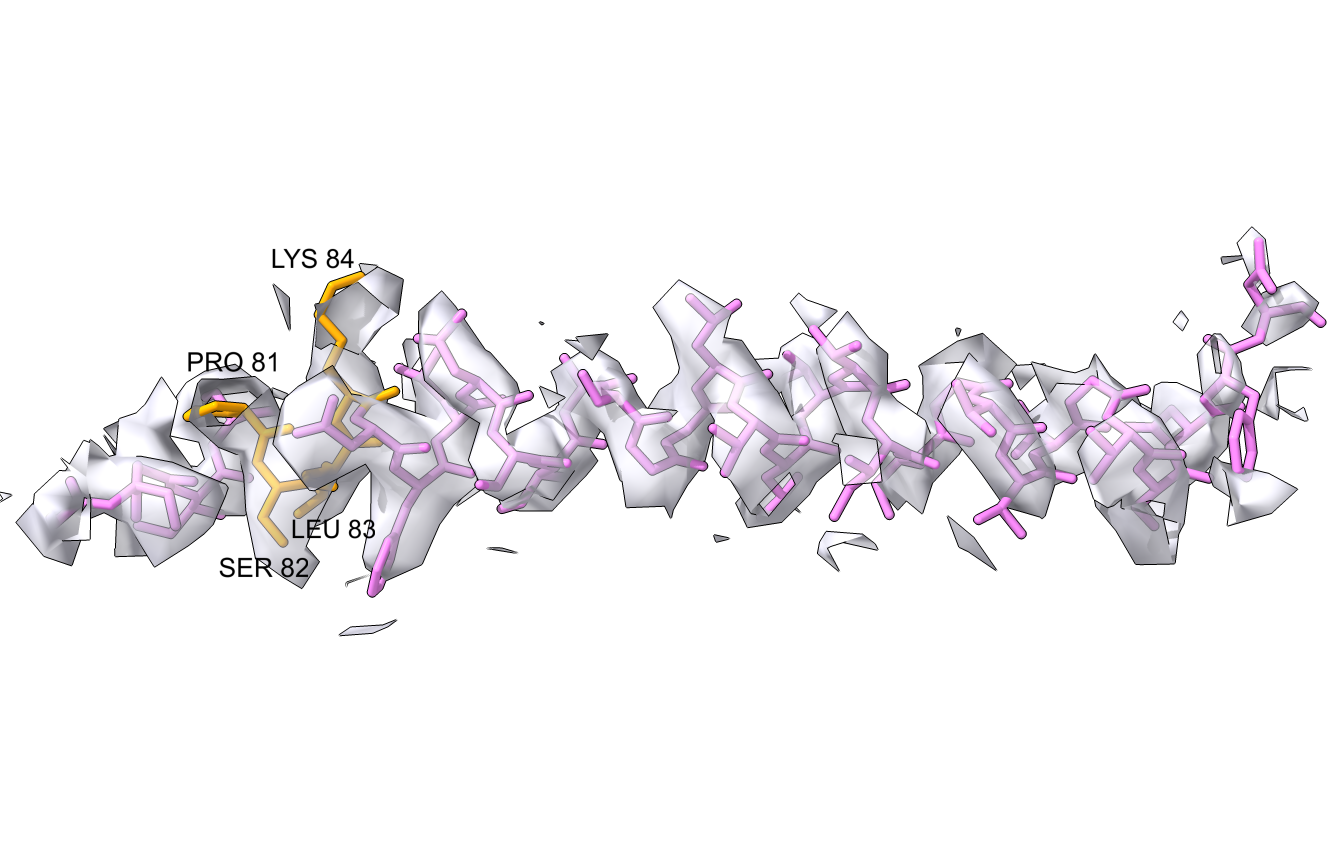

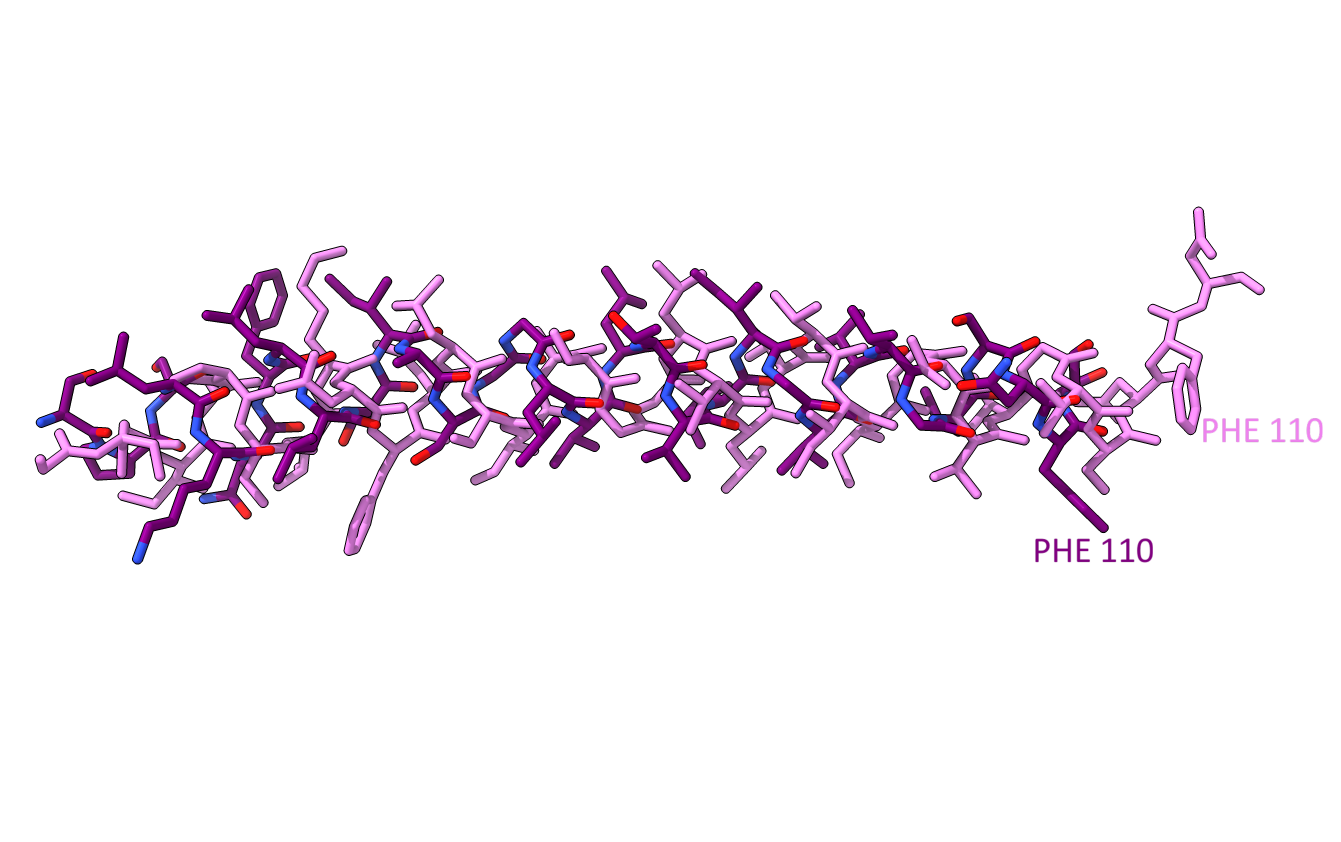


7OUI

5MDX

**A**

**B**

Figure S9 – PsbX: A, representation of the shift between PsbX subunit in our EM model (7OUI, pink) and the 5MDX model (purple); B, the PsbX subunit fit into its respective density of our EM map.


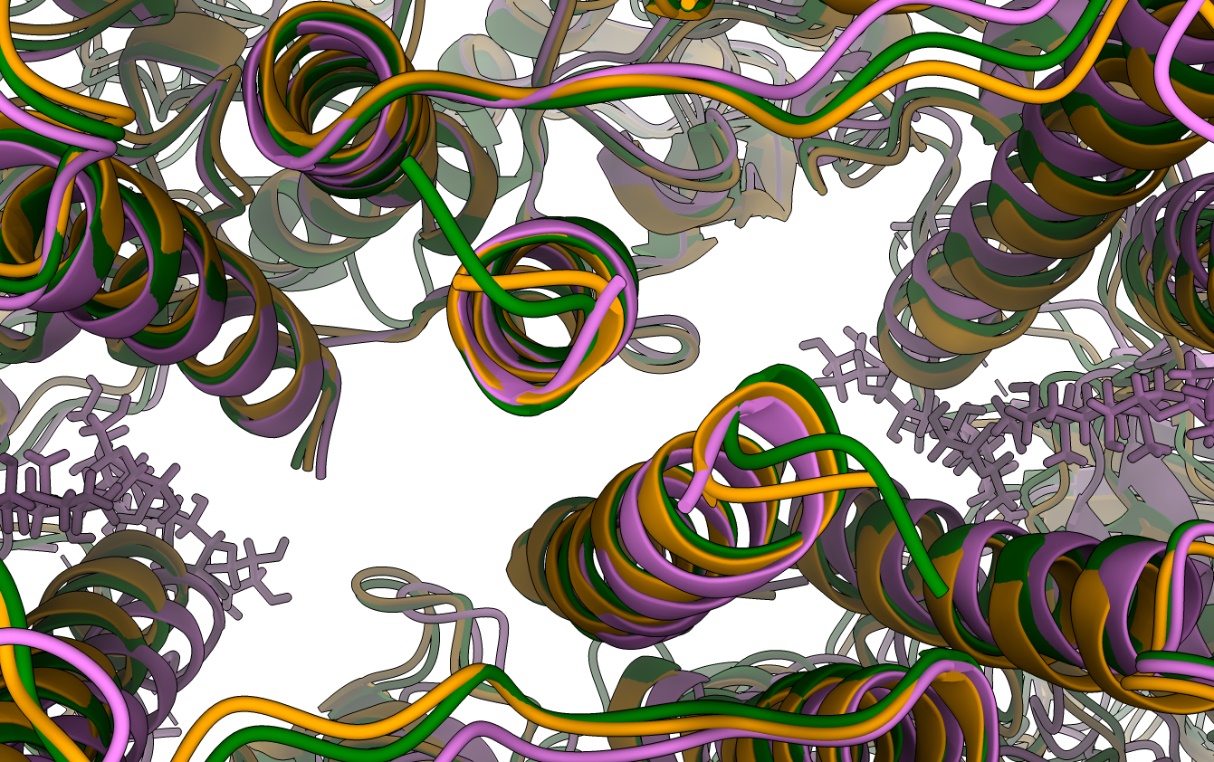


Figure S10 - Dimerization interface between monomeric PSII cores: 3JCU in green, 5XNM in orange, 7OUI in violet. At the dimerization axis (represented in blue dashed line), two digitonin molecules can be found on model 7OUI. Models aligned to the PsbM protein of the same monomer (above the dimerization interface). The transmembrane helices highlighted in yellow indicate the secondary structure motifs to which intermonomer Cα-Cα atomic distances where calculated (see Figure S11) and the blue dashed lines represent the dimerization axis of the PSII complex.


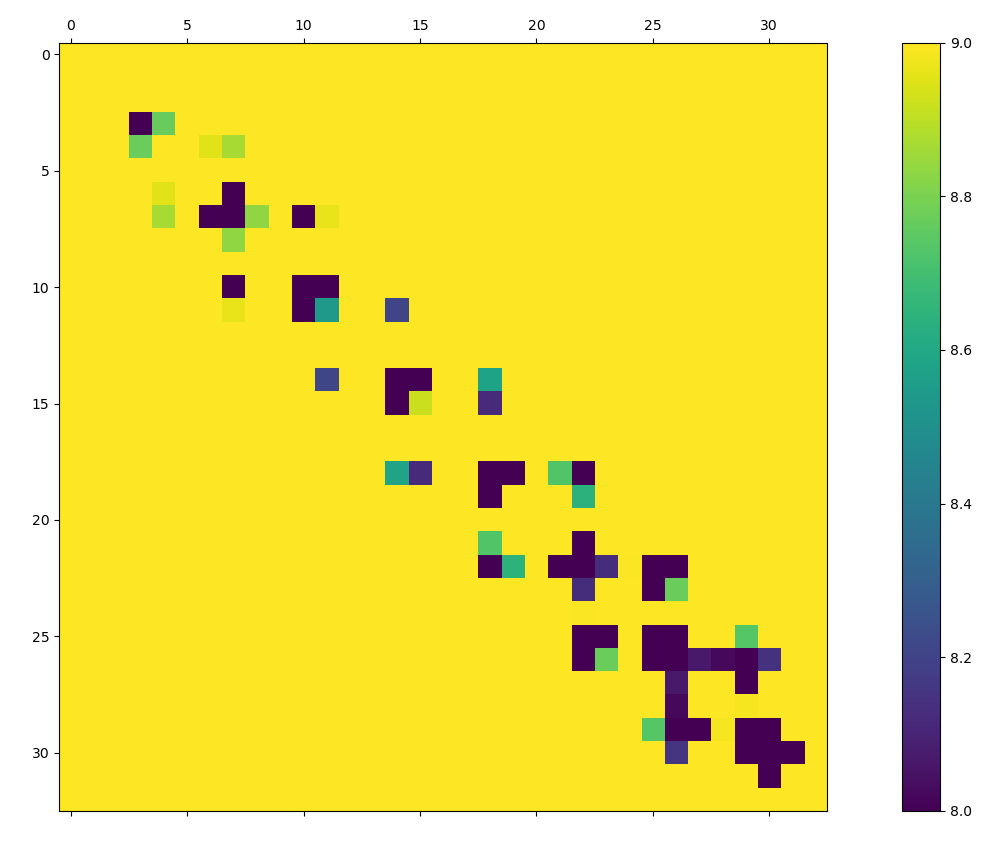


0

30

15


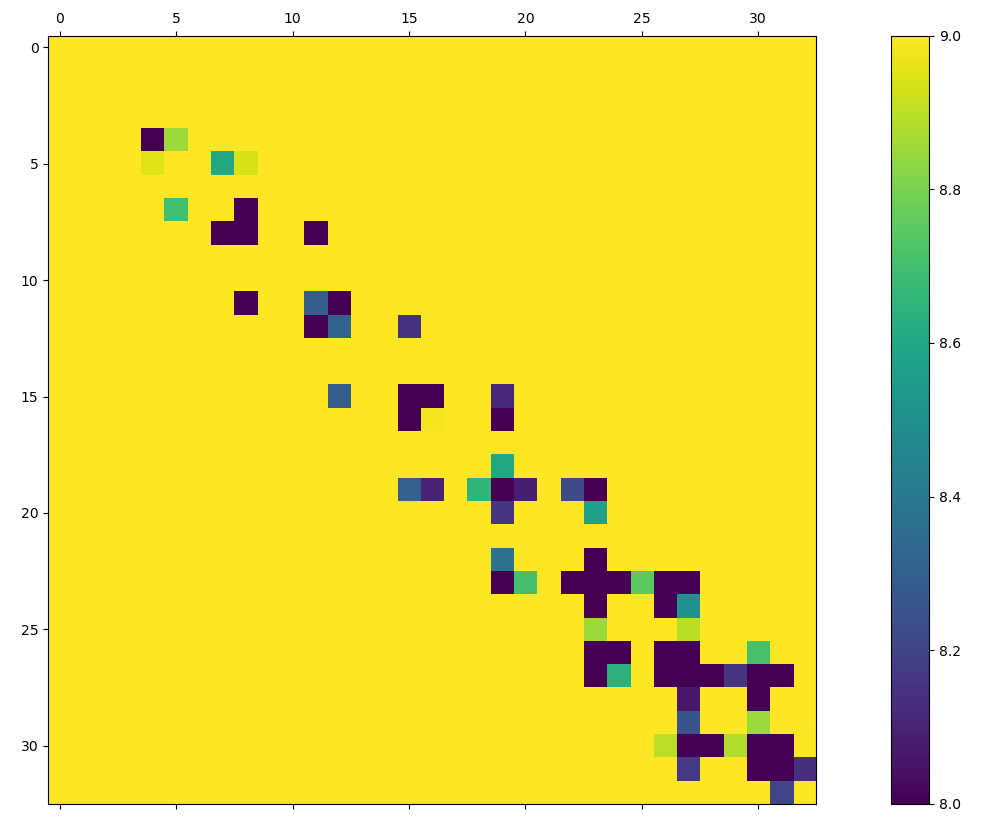


0

30

15


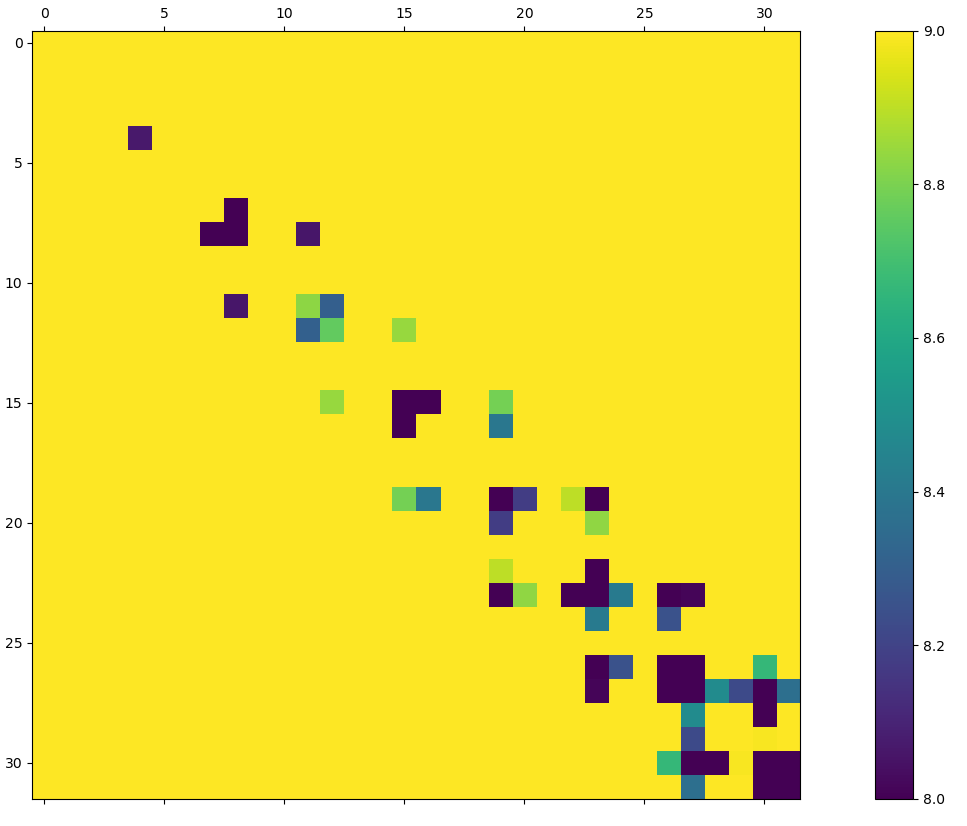


0

30

15

9

8

Inter-atomic distance (Å)

**5XNM**

**3JCU**

**7OUI**

**Residue number**

30

15

0

**Residue number**

**Residue number**

**Residue number**

Figure S11 - Heatmaps reporting the calculated intermonomer Cα-Cα atomic distances, for each high-resolution higher plant PSII model, between the small PsbM proteins from opposite PSII monomers—highlighted in yellow in figure S10.


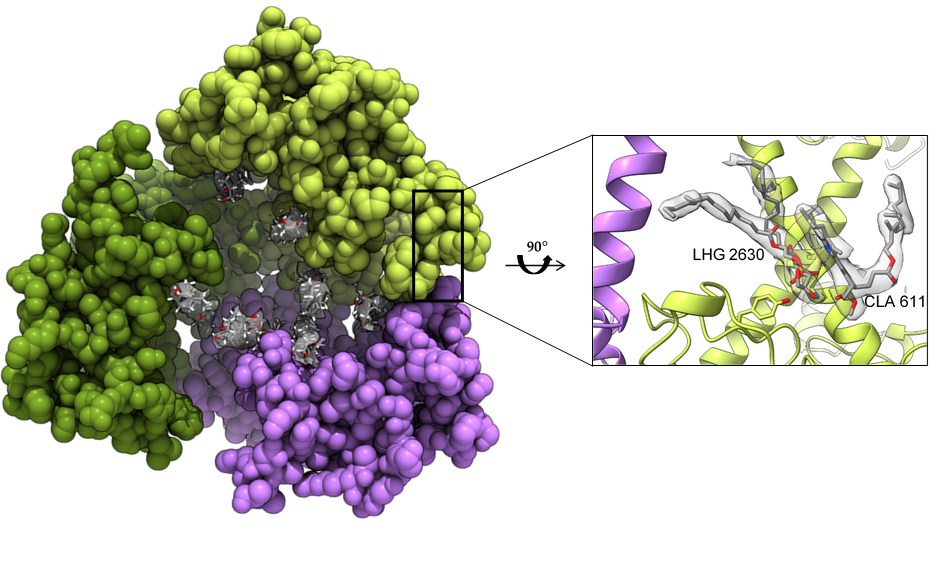
Figure S12 – Digitonin molecules occupy the center of the LHCII trimer. Inset shows the binding site of the PG lipid LHG2630 and chlorophyll a CLA611, bound to the monomer S-LHCIIA. The residues Tyr78 and Lys217 of S-LHCIIA participating in the binding of LHG2630 are displayed.


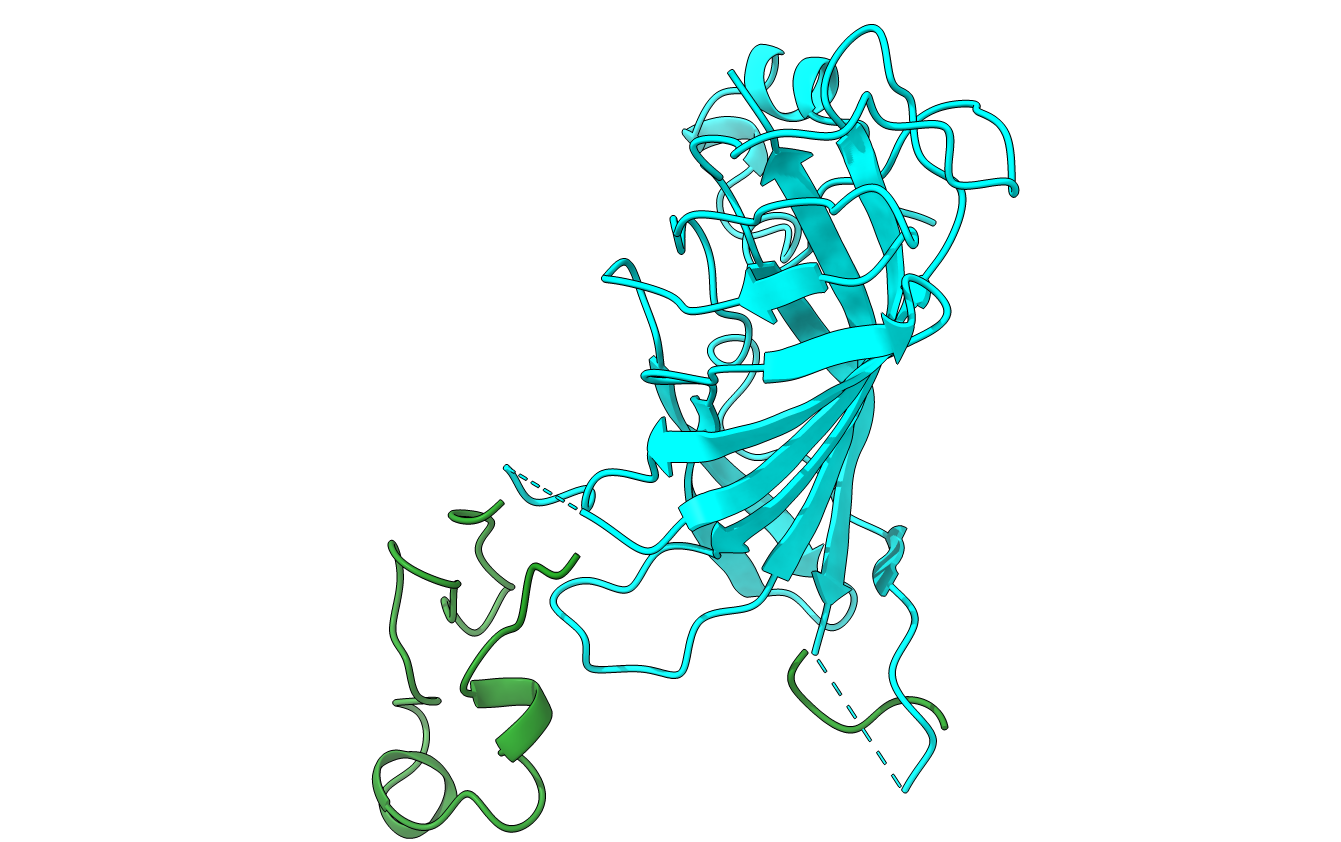


Short loop

Long loop

7OUI

3JCU

**PsbO**

Asn 153

ALA 147

GLY 238

ALA 280

Figure S13 – Representation of PsbO long- and short- loop interruptions (in cyan) and the peptides that constitute the same loops in spinach PSII (3JCU, in green). The last modelled amino acids at N- and C- termini of both Arabidopsis loops. Are represented with explicit labels.

***Pisum sativum***

***Spinacia oleracea***

***Arabidopsis thaliana***

Conservation

Quality

Consensus

***Pisum sativum***

***Spinacia oleracea***

***Arabidopsis thaliana***

Conservation

Quality

Consensus

***Pisum sativum***

***Spinacia oleracea***

***Arabidopsis thaliana***

Conservation

Quality

Consensus


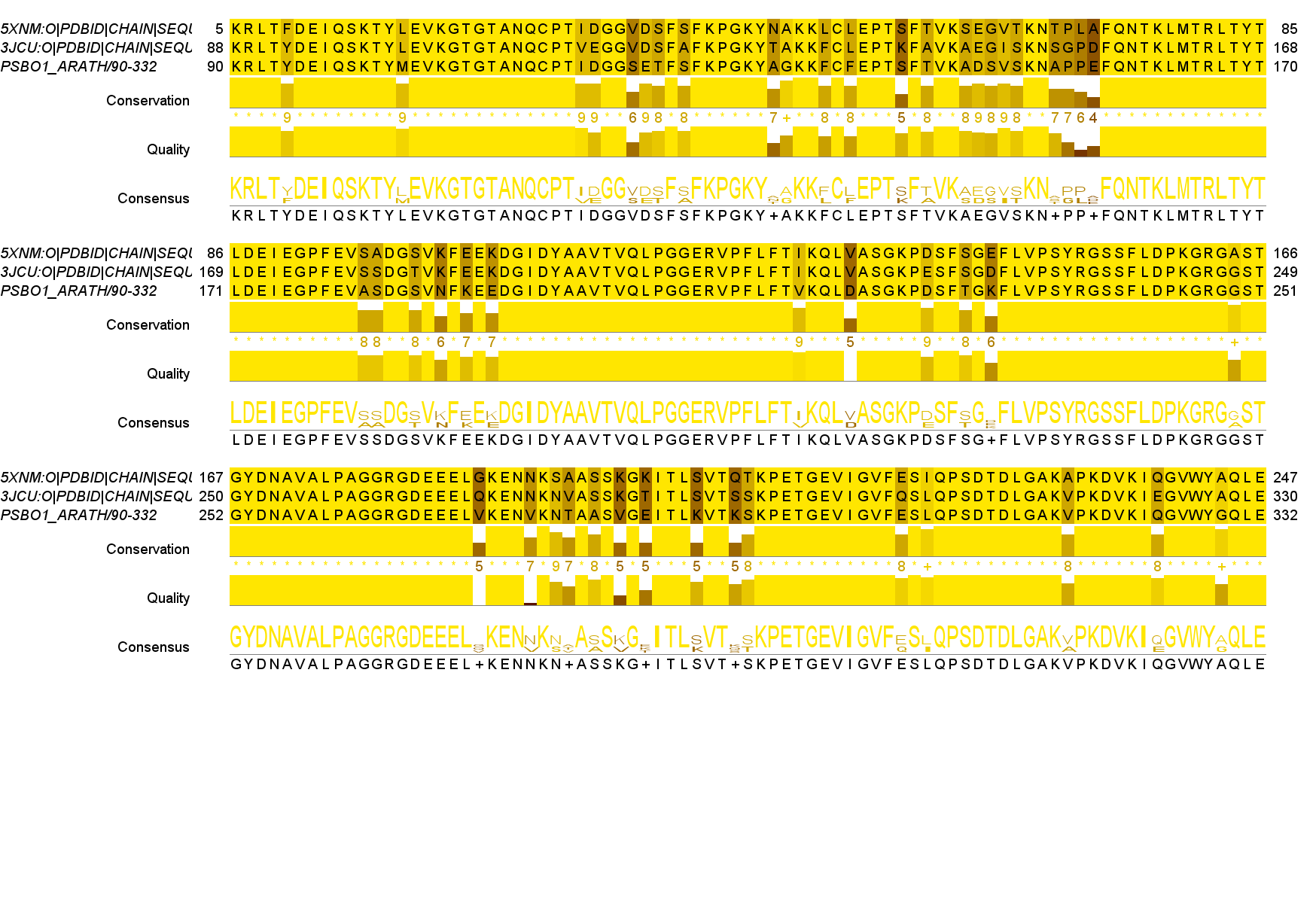


**Short-loop**

**Long-loop**

Figure S14 - Sequence alignment of the PsbO proteins of different higher plants.

ALA 147


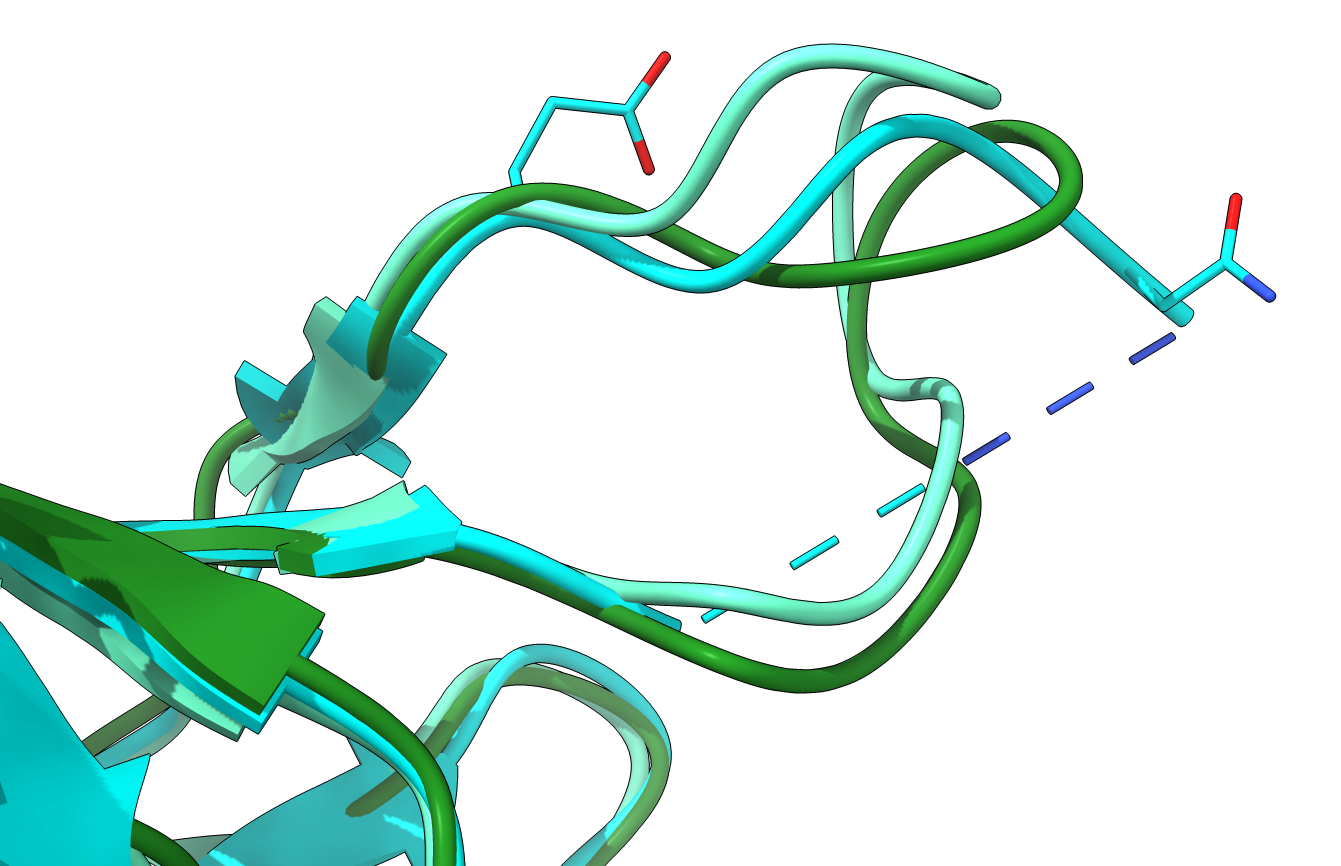


ASN 153

GLU 157

**Short loop**

7OUI

5XNM

3JCU


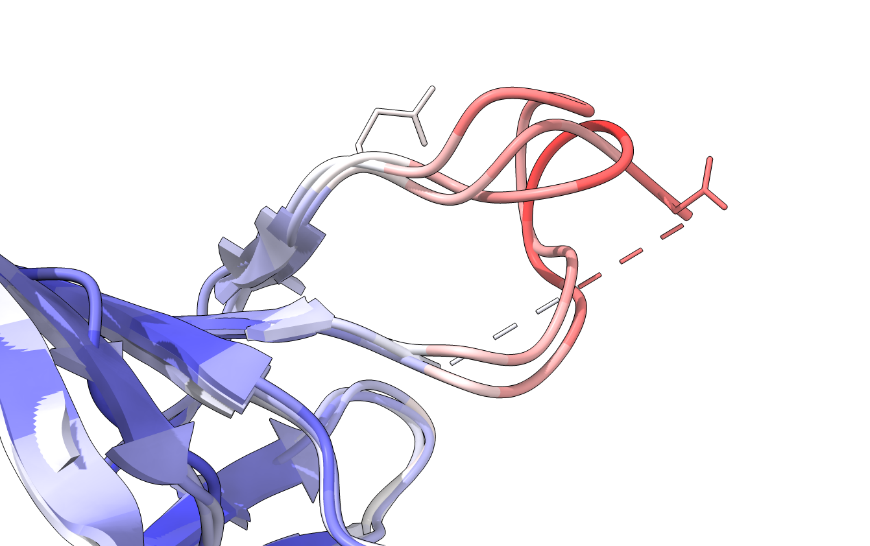


Figure S15 - The PsbO short-loop missing in Arabidopsis PSII model is in a region of several non-conserved amino acids (position 153-157, according to Arabidopsis numbering). In accordance with 5XNM and 3JCU models, which represent the missing amino acids in 7OUI, all the amino acids in the loop region have a high b-factor, factors which likely justify the flexibility and difficulty to model such loop in Arabidopsis.


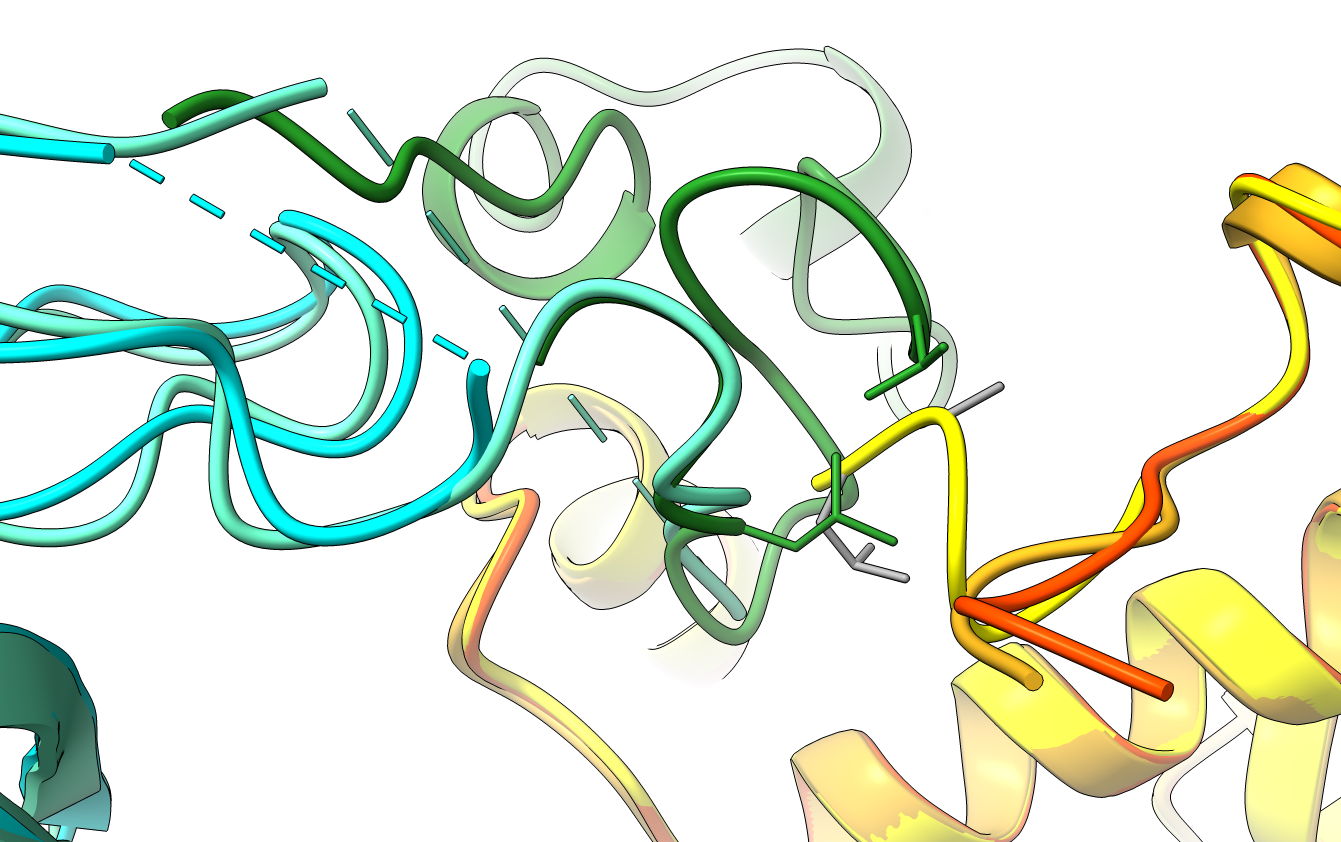


**PsbO**

7OUI **PsbO** / **D2**

5XNM **PsbO** / **D2**

3JCU **PsbO long-loop** / **D2**

**D2**

D2 C-terminus

Figure S16 – Representation of the PsbO protein long-loop and the D2 C-terminus of the different higher plants PSII, with a different conformation for Arabidopsis PSII. Such twist occurs at the peptide bond Gly350-Asn351, conflicting with original location of PsbO long-loop.
