# Supplementary material for: High-resolution model of Arabidopsis Photosystem II reveals the structural consequences of digitonin-extraction
Source: Sci Rep. 2021 Jul 30;11:15534. doi: 10.1038/s41598-021-94914-x (PMC8324835; doi:10.1038/s41598-021-94914-x)
Supplement: Supplementary file 2 — Supplementary Information 2. [file 41598_2021_94914_MOESM2_ESM.docx]

*Table S1 - Statistics of cryo-EM data and structural analysis of the C2S2M2-type PSII supercomplex refined at 2.8 Å resolution.*

**PSII C_2_S_2_M_2_ (EMD-13078) (PDB 7OUI)**

**Data collection and processing**

Hardware

Microscope Titan Krios

Detector (mode) Gatan K2 BioQuantum (counted)

Voltage (keV) 300

Spherical aberration 2.7

Magnification 165 000 ×

Electron exposure (e/Å^2^) 59.7

Defocus range (μm) -1.5 to -3.0

Pixel size (Å) 0.82

Symmetry imposed C2

Initial particle images (no.) 416 262

Final particle images (no.) 110 659

Map resolution (Å) 2.79 (C_2_S_2_ masked) / 3.13 (C_2_S_2_M_2_)

FSC threshold 0.143

**Refinement**

Initial models used (PDB code) 5MDX and 5XNM

Model composition

Non-hydrogen atoms 85897

Protein residues 8342

Ligands 374

Water molecules 139

B factors (Å^2^)

Protein

Ligand

R.m.s. deviations

Bond lengths (Å)

Bond angles (°)

Validation

MolProbity score 2.75

Clash score 51.35

Poor rotamers (%)

Ramachandran plot

Favored (%) 94.70

Allowed (%) 5.13

Outliers (%) 0.17

*Table S2 - Details on the modelled PSII core subunits. The most common protein names can be found in bold, while alternative names are contained within parenthesis. The molecular weight and pI—computed using ProtParam tool from the Expasy server—regarding the full sequences of mature proteins (according with the respective UniProt entries, accession code preceded by a greater-than symbol before the sequence). The sequences have amino acids letters coloured in 2 distinct colours:* ***black****, all the amino acids modelled in #XXX;* ***red****, amino acids yet unobserved/undistinguishable in a 3D map of* Arabidopsis thaliana*. Modelling completeness is the proportion of modelled in amino acid in #XXX with respect to the mature protein sequence.*

| **Chain ID** | **Protein Name** | **Gene name** | **Molecular weight**  **(kDa)** | **pI** | **Sequence (mature protein)** | **Modelling completeness**  **(%)** | **Model fit into the electrostatic potential map** |
| --- | --- | --- | --- | --- | --- | --- | --- |
| A,a | **D1**  (Q_B_ protein) | PSBA | 37.98 | 5.21 | >P83755  TAILERRESESLWGRFCNWITSTENRLYIGWFGVLMIPTLLTATSVFIIAFIAAPPVDIDGIREPVSGSLLYGNNIISGAIIPTSAAIGLHFYPIWEAASVDEWLYNGGPYELIVLHFLLGVACYMGREWELSFRLGMRPWIAVAYSAPVAAATAVFLIYPIGQGSFSDGMPLGISGTFNFMIVFQAEHNILMHPFHMLGVAGVFGGSLFSAMHGSLVTSSLIRETTENESANEGYRFGQEEETYNIVAAHGYFGRLIFQYASFNNSRSLHFFLAAWPVVGIWFTALGISTMAFNLNGFNFNQSVVDSQGRVINTWADIINRANLGMEVMHERNAHNFPLDLA | 98.8 | 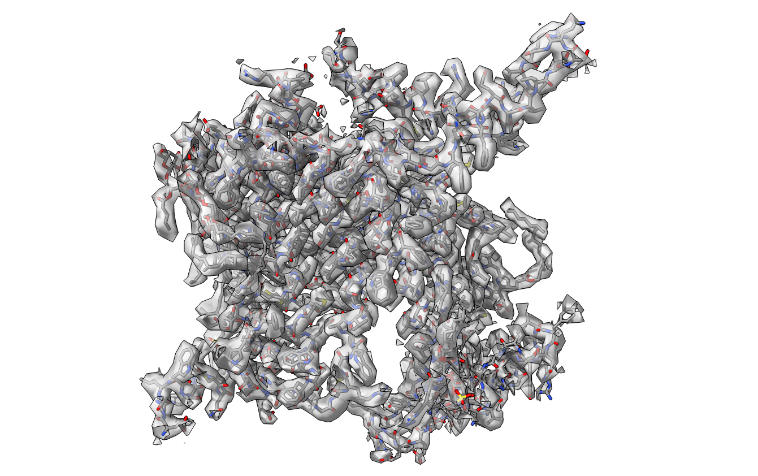 |
| B,b | **CP47** | PSBB | 56.04 | 6.40 | >P56777  MGLPWYRVHTVVLNDPGRLLAVHIMHTALVAGWAGSMALYELAVFDPSDPVLDPMWRQGMFVIPFMTRLGITNSWGGWNITGGTITNPGLWSYEGVAGAHIVFSGLCFLAAIWHWVYWDLEIFCDERTGKPSLDLPKIFGIHLFLSGVACFGFGAFHVTGLYGPGIWVSDPYGLTGKVQPVNPAWGVEGFDPFVPGGIASHHIAAGTLGILAGLFHLSVRPPQRLYKGLRMGNIETVLSSSIAAVFFAAFVVAGTMWYGSATTPIELFGPTRYQWDQGYFQQEIYRRVSAGLAENQSLSEAWAKIPEKLAFYDYIGNNPAKGGLFRAGSMDNGDGIAVGWLGHPVFRNKEGRELFVRRMPTFFETFPVVLVDGDGIVRADVPFRRAESKYSVEQVGVTVEFYGGELNGVSYSDPATVKKYARRAQLGEIFELDRATLKSDGVFRSSPRGWFTFGHASFALLFFFGHIWHGARTLFRDVFAGIDPDLDAQVEFGAFQKLGDPTTKRQAV | 95.9 | 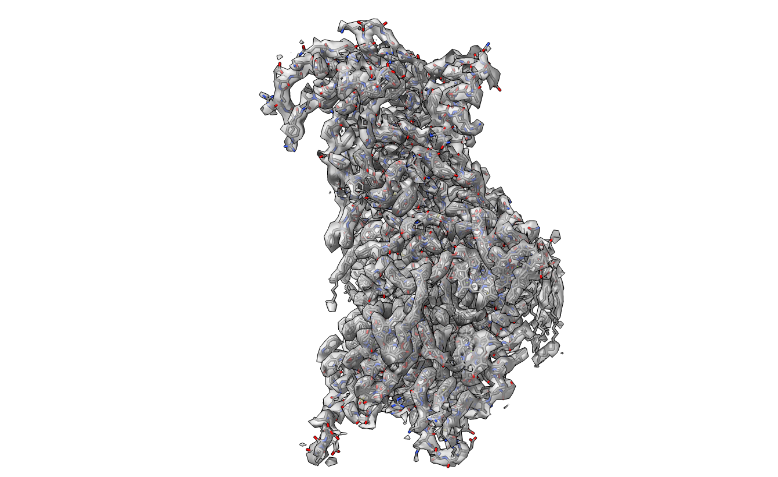 |
| C,c | **CP43** | PSBC | 50.04 | 6.34 | >P56778  TLFNGTLALAGRDQETTGFAWWAGNARLINLSGKLLGAHVAHAGLIVFWAGAMNLFEVAHFVPEKPMYEQGLILLPHLATLGWGVGPGGEVIDTFPYFVSGVLHLISSAVLGFGGIYHALLGPETLEESFPFFGYVWKDRNKMTTILGIHLILLGVGAFLLVFKALYFGGVYDTWAPGGGDVRKITNLTLSPSVIFGYLLKSPFGGEGWIVSVDDLEDIIGGHVWLGSICIFGGIWHILTKPFAWARRALVWSGEAYLSYSLAALSVCGFIACCFVWFNNTAYPSEFYGPTGPEASQAQAFTFLVRDQRLGANVGSAQGPTGLGKYLMRSPTGEVIFGGETMRFWDLRAPWLEPLRGPNGLDLSRLKKDIQPWQERRSAEYMTHAPLGSLNSVGGVATEINAVNYVSPRSWLSTSHFVLGFFLFVGHLWHAGRARAAAAGFEKGIDRDFEPVLSMTPLN | 94.3 | 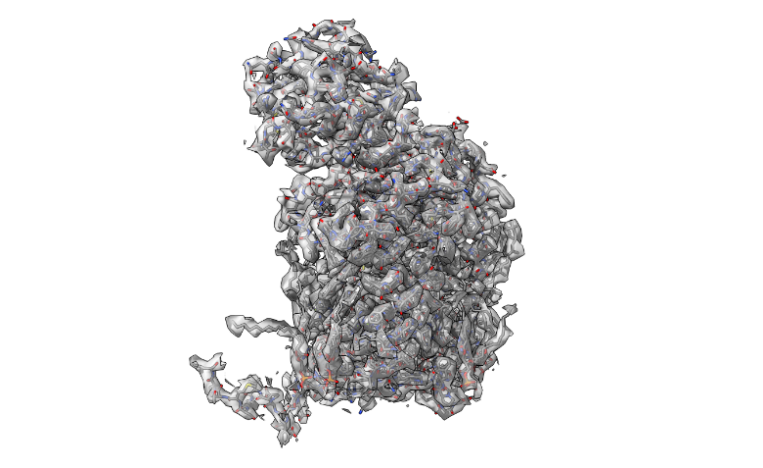 |
| D,d | **D2**  (Q_A_ protein) | PSBD | 39.42 | 5.46 | >P56761  TIALGKFTKDEKDLFDIMDDWLRRDRFVFVGWSGLLLFPCAYFALGGWFTGTTFVTSWYTHGLASSYLEGCNFLTAAVSTPANSLAHSLLLLWGPEAQGDFTRWCQLGGLWAFVALHGAFALIGFMLRQFELARSVQLRPYNAIAFSGPIAVFVSVFLIYPLGQSGWFFAPSFGVAAIFRFILFFQGFHNWTLNPFHMMGVAGVLGAALLCAIHGATVENTLFEDGDGANTFRAFNPTQAEETYSMVTANRFWSQIFGVAFSNKRWLHFFMLFVPVTGLWMSALGVVGLALNLRAYDFVSQEIRAAEDPEFETFYTKNILLNEGIRAWMAAQDQPHENLIFPEEVLPRGNAL | 97.2 | 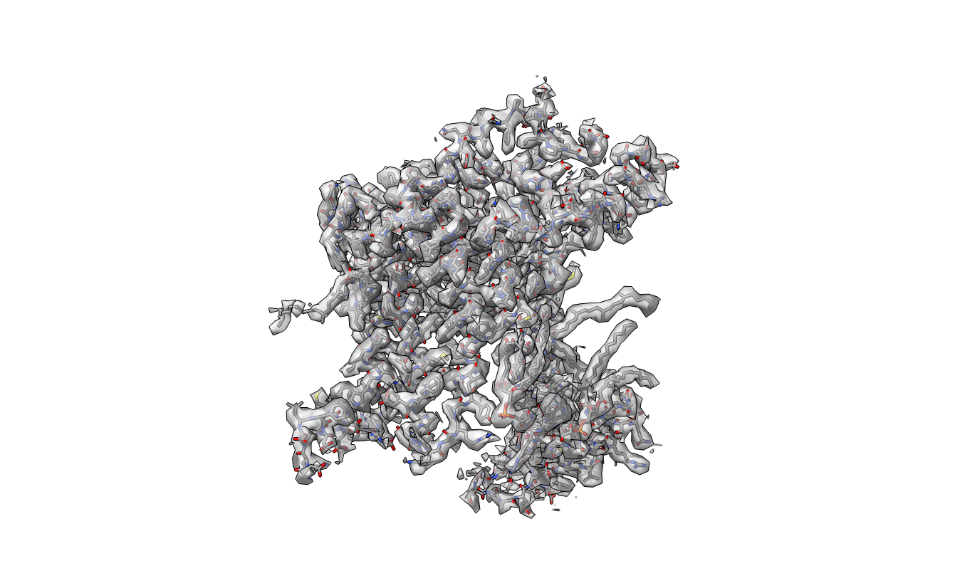 |
| E,e | **Cytochrome b_559_α** | PSBE | 9.39 | 4.83 | >P56779  MSGSTGERSFADIITSIRYWVIHSITIPSLFIAGWLFVSTGLAYDVFGSPRPNEYFTESRQGIPLITGRFDSLEQLDEFSRSF | 79.5 | 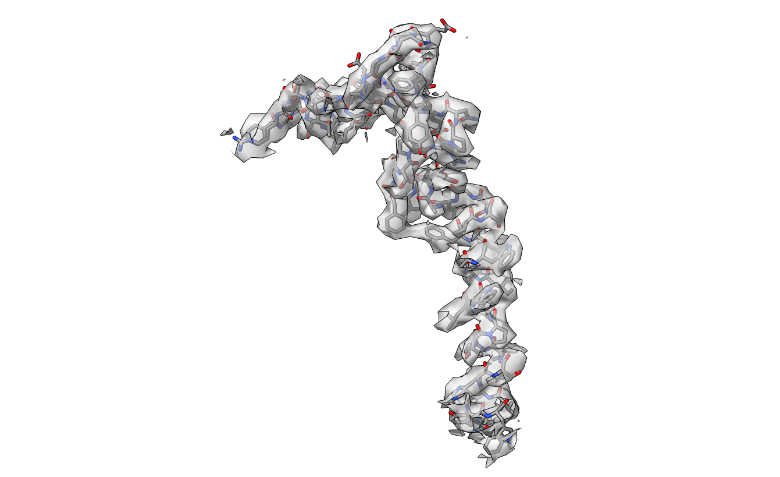 |
| F,f | **Cytochromeb_559_β** | PSBF | 4.42 | 10.74 | >P62095  MTIDRTYPIFTVRWLAVHGLAVPTVSFLGSISAMQFIQR | 74.4 | 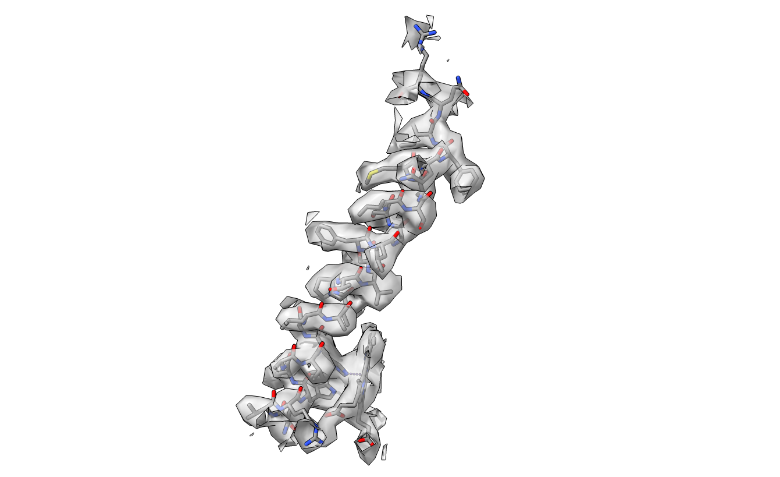 |
| H,h | **Phosphoprotein H**  (PSII-H, 10 kDa phosphoprotein) | PSBH | 7.57 | 6.32 | >P56780  ATQTVEDSSRSGPRSTTVGKLLKPLNSEYGKVAPGWGTTPLMGVAMALFAVFLSIILEIYNSSVLLDGISVN | 83.3 | 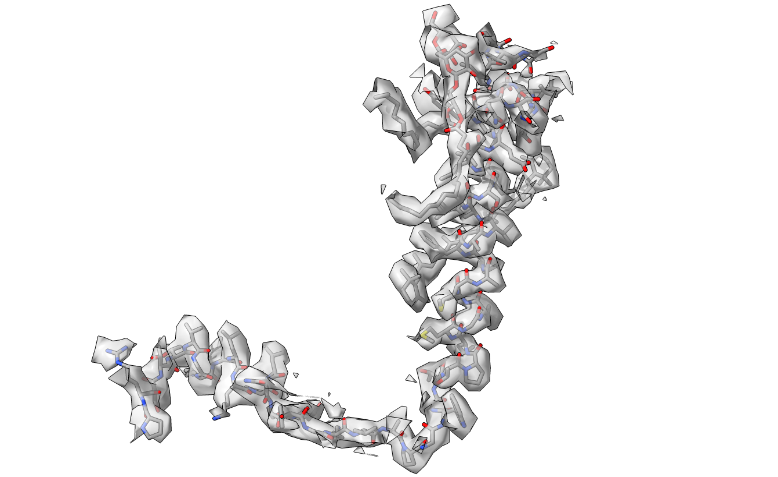 |
| I,i | **Protein I**  (PSII-I, 4.8 kDa protein) | PSBI | 4.17 | 5.94 | >P62100  MLTLKLFVYTVVIFFVSLFIFGFLSNDPGRNPGREE | 97.2 | 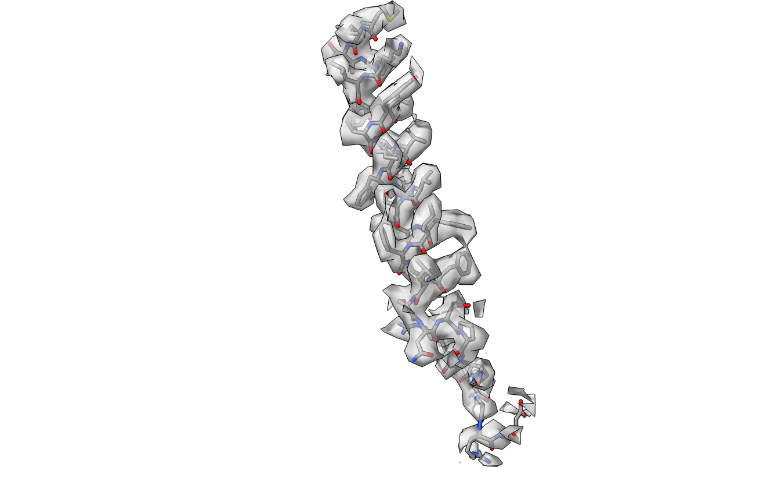 |
| K,k | **Protein K**  (PSII-K) | PSBK | 4.24 | 6.07 | >P56782  KLPEAYAFLNPIVDVMPVIPLFFLLLAFVWQAAVSFR | 100.0 | 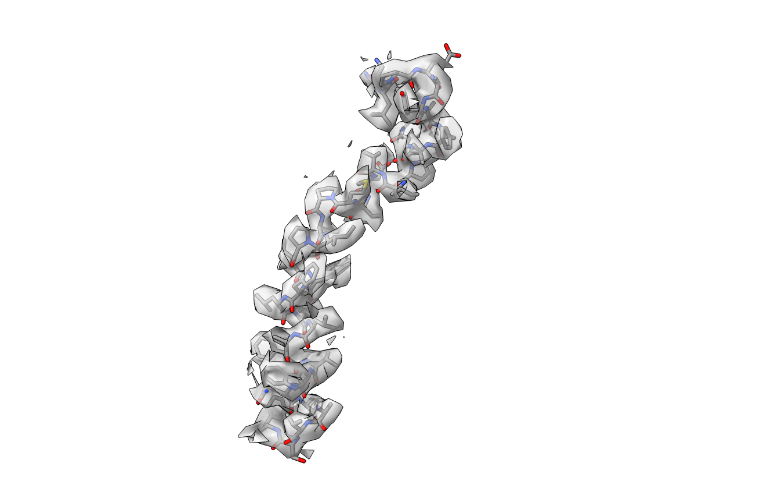 |
| L,l | **Protein L** (PSII-L) | PSBL | 4.47 | 4.53 | >P60129  MTQSNPNEQSVELNRTSLYWGLLLIFVLAVLFSNYFFN | 94.7 | 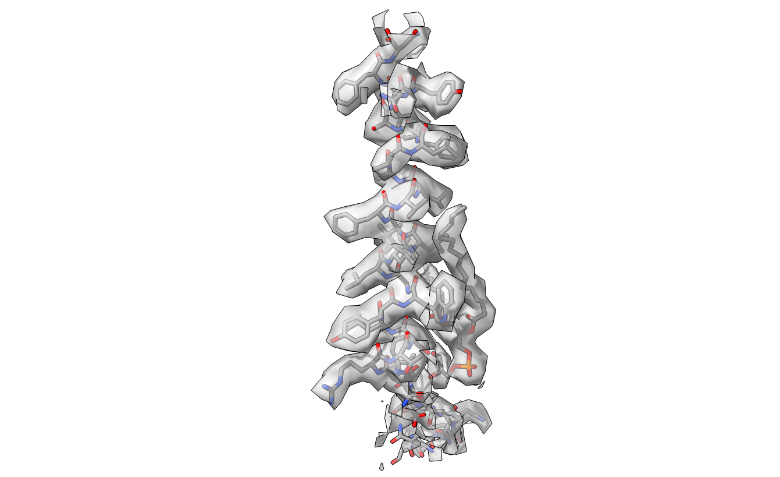 |
| M,m | **Protein M** (PSII-M) | PSBM | 3.78 | 4.37 | >P62109  MEVNILAFIATALFILVPTAFLLIIYVKTVSQND | 94.12 | 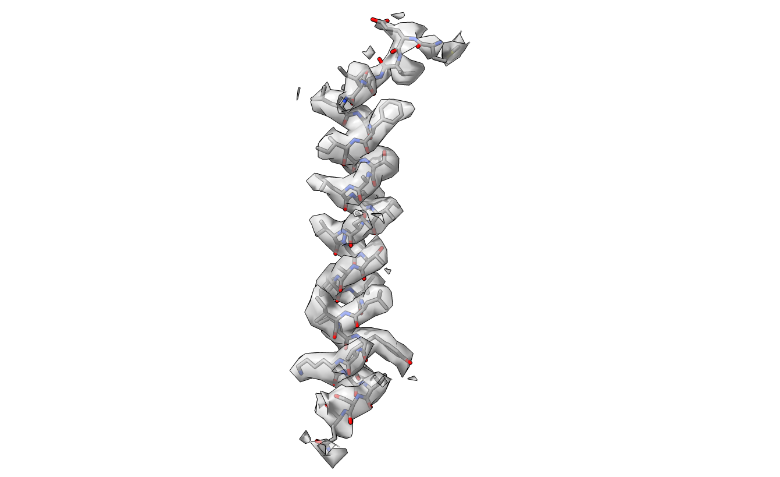 |
| O,o | **Oxygen-evolving enhancer protein 1-1**, (OEE1, 33 kDa Manganese-stabilizing protein 1, MSP-1, OEC 33 kDa subunit) | PSBO1 | 26.57 | 4.93 | >P23321  EGAPKRLTYDEIQSKTYMEVKGTGTANQCPTIDGGSETFSFKPGKYAGKKFCFEPTSFTVKADSVSKNAPPEFQNTKLMTRLTYTLDEIEGPFEVASDGSVNFKEEDGIDYAAVTVQLPGGERVPFLFTVKQLDASGKPDSFTGKFLVPSYRGSSFLDPKGRGGSTGYDNAVALPAGGRGDEEELVKENVKNTAASVGEITLKVTKSKPETGEVIGVFESLQPSDTDLGAKVPKDVKIQGVWYGQLE | 79.8 | 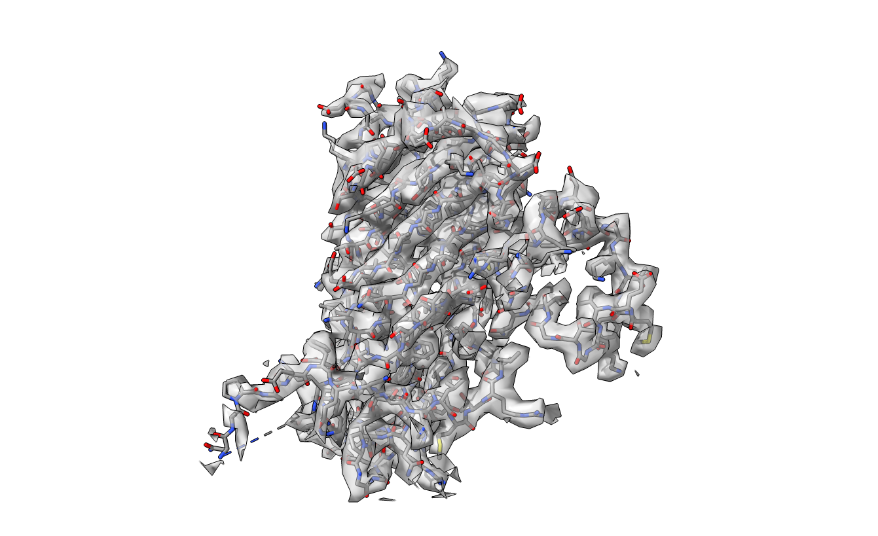 |
| T,t | **Protein Tc** (PSII-T)  (Chloroplast T) | PSBTC | 3.82 | 9.52 | >P61839  MEALVYTFLLVSTLGIIFFAIFFREPPKISTKK | 87.9 | 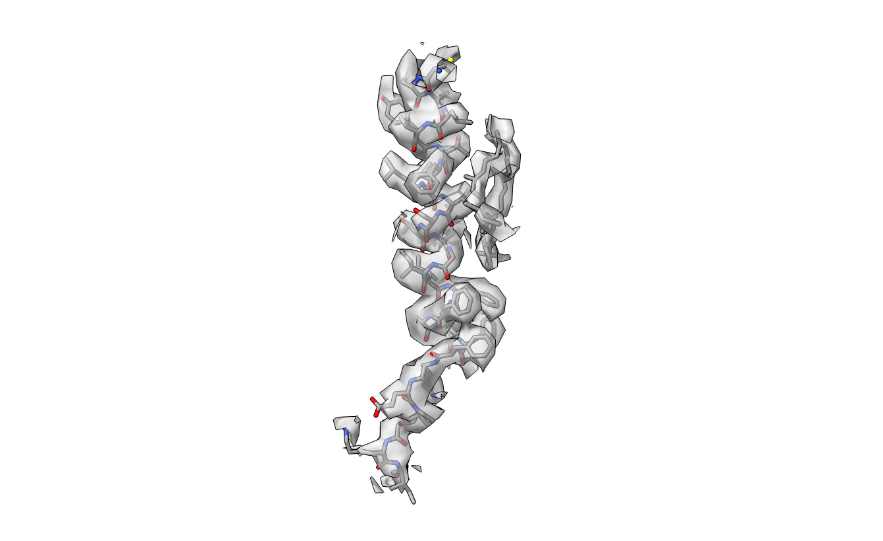 |
| U,u | **Protein Tn** (PSII-T)  (nucleus T, Extrinsic T) | PSBTN | 3.17 | 9.70 | >Q39195  EPKRGTEAAKKKYAQVCVTMPTAKICRY | 89.3 | 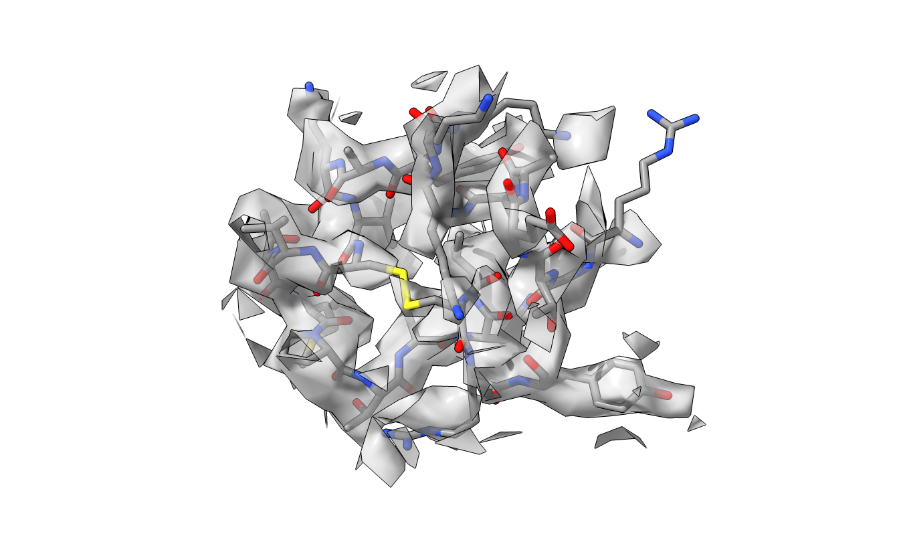 |
| W,w | **Protein W**  (PSII-W, PSII 6.1 kDa protein) | PSBW | 6.04 | 3.67 | >Q39194  LVDERMSTEGTGLPFGLSNNLLGWILFGVFGLIWTFFFVYTSSLEEDEESGLSL | 100.0 | 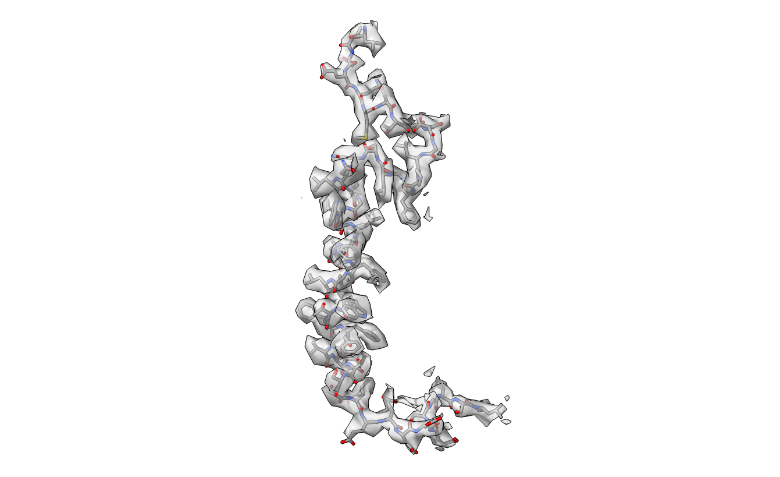 |
| X,x | **Protein X**  (PSII-X) | PSBX | 4.18 | 9.99 | >Q9SKI3  AGSGISPSLKNFLLSIASGGLVLTVIIGVVVGVSNFDPVKRT | 85.7 | 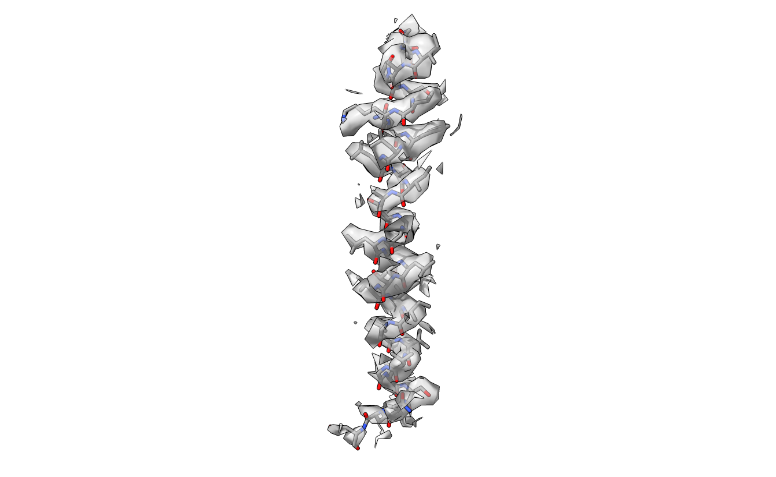 |
| Z,z | **Protein Z** (PSII-Z) | PSBZ, YCF9 | 6.57 | 5.59 | >P56790  MTIAFQLAVFALIITSSILLISVPVVFASPDGWSSNKNVVFSGTSLWIGLVFLVGILNSLIS | 100.0 | 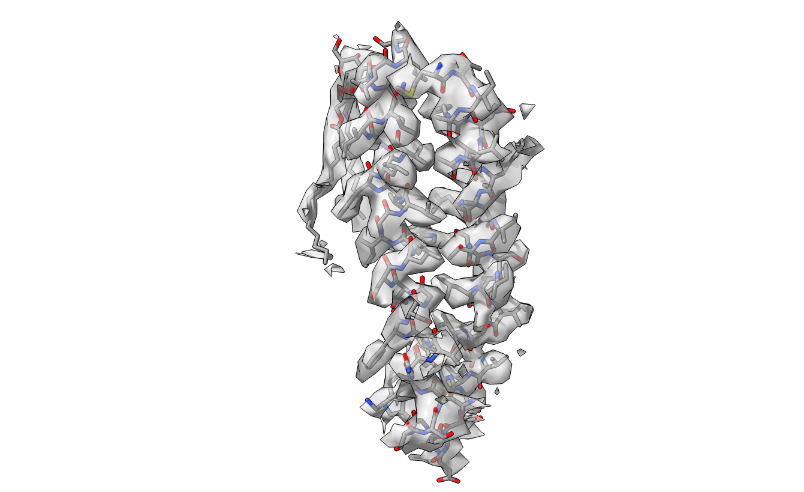 |

*Table S3 - Details on the modelled PSII minor and major antennas. The most common protein names can be found in bold, while alternative names are contained within parenthesis. The molecular weight and pI—computed using ProtParam tool from the Expasy server—regarding the full sequences of mature proteins (according with the respective UniProt entries). The protein sequences presented correspond exclusively to the amino acids present in our model.*

| **Chain ID** | **Protein Name** | **Gene name** | **Molecular weight**  **(kDa)** | **pI** | **Sequence of modelled protein** | **Model fit into the electrostatic potential map** |
| --- | --- | --- | --- | --- | --- | --- |
| G,g | **Chlorophyll a-b binding protein 1**  (Chlorophyll a-b protein 140, CAB-140, LHCII type I CAB-1)  **S-Trimer** | LHCB1.3 | 24.86 | 5.12 | >P04778  GSPWYGSDRVKYLGPFSGESPSYLTGEFPGDYGWDTAGLSADPETFARNRELEVIHSRWAMLGALGCVFPELLARNGVKFGEAVWFKAGSQIFSDGGLDYLGNPSLVHAQSILAIWATQVILMGAVEGYRVAGNGPLGEAEDLLYPGGSFDPLGLATDPEAFAELKVKELKNGRLAMFSMFGFFVQAIVTGKGPIENLADHLADPV | 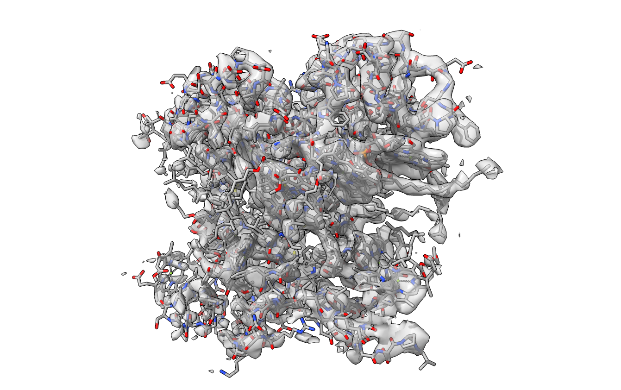 |
| N,n |  |  |  |  | >P04778  SPWYGSDRVKYLGPFSGESPSYLTGEFPGDYGWDTAGLSADPETFARNRELEVIHSRWAMLGALGCVFPELLARNGVKFGEAVWFKAGSQIFSDGGLDYLGNPSLVHAQSILAIWATQVILMGAVEGYRVAGNGPLGEAEDLLYPGGSFDPLGLATDPEAFAELKVKELKNGRLAMFSMFGFFVQAIVTGKGPIENLADHLA | 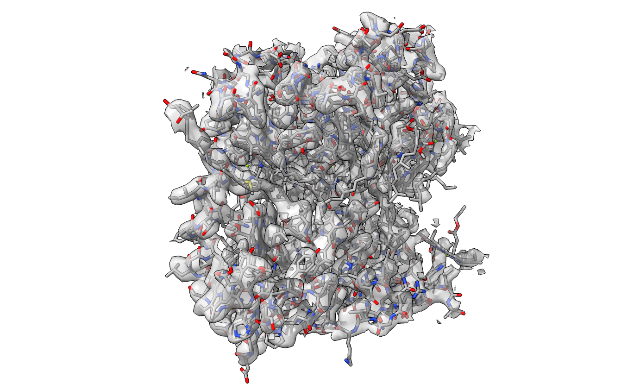 |
| Y,y |  |  |  |  | >P04778  GSPWYGSDRVKYLGPFSGESPSYLTGEFPGDYGWDTAGLSADPETFARNRELEVIHSRWAMLGALGCVFPELLARNGVKFGEAVWFKAGSQIFSDGGLDYLGNPSLVHAQSILAIWATQVILMGAVEGYRVAGNGPLGEAEDLLYPGGSFDPLGLATDPEAFAELKVKELKNGRLAMFSMFGFFVQAIVTGKGPIENLADHLADPVNNNAWAF | 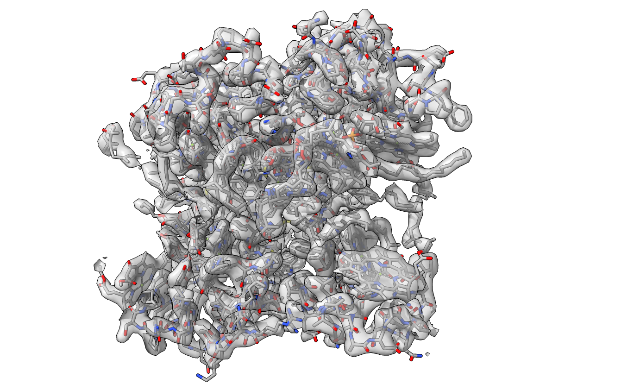 |
| 1,5 | **Chlorophyll a-b binding protein 1**  **M-Trimer** | LHCB1.4 | 23.67 | 4.63 | > Q39142  SPWYGSDRVKYLGPFSGEPPSYLTGEFPGDYGWDTAGLSADPETFARNRELEVIHSRWAMLGALGCVFPELLARNGVKFGEAVWFKAGSQIFSDGGLDYLGNPSLVHAQSILAIWATQVILMGAVEGYRVAGDGPLGEAEDLLYPGGSFDPLGLATDPEAFAELKVKELKNGRLAMFSMFGFFVQAIVTGKGPLENLADHLA | 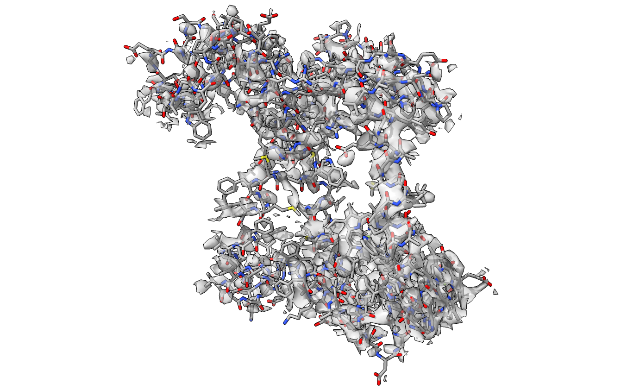 |
| 2,6 | **Chlorophyll a-b binding protein 3**  (LHCB3*1)  **M-Trimer** | LHCB3 | 26.43 | 4.85 | >Q9S7M0  DLWYGPDRVKYLGPFSVQTPSYLTGEFPGDYGWDTAGLSADPEAFAKNRALEVIHGRWAMLGAFGCITPEVLQKWVRVDFKEPVWFKAGSQIFSEGGLDYLGNPNLVHAQSILAVLGFQVILMGLVEGFRINGLDGVGEGNDLYPGGQYFDPLGLADDPVTFAELKVKEIKNGRLAMFSMFGFFVQAIVTGKGPLENLLDHLDNP | 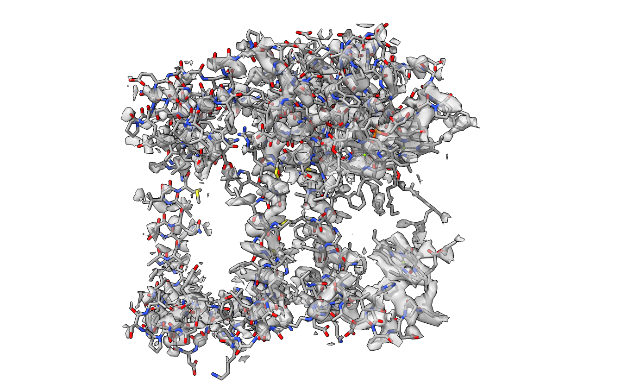 |
| 3,7 | **Chlorophyll a-b binding protein 1**  **M-Trimer** | LHCB1.4 | 23.67 | 4.63 | > Q39142  SPWYGSDRVKYLGPFSGEPPSYLTGEFPGDYGWDTAGLSADPETFARNRELEVIHSRWAMLGALGCVFPELLARNGVKFGEAVWFKAGSQIFSDGGLDYLGNPSLVHAQSILAIWATQVILMGAVEGYRVAGDGPLGEAEDLLYPGGSFDPLGLATDPEAFAELKVKELKNGRLAMFSMFGFFVQAIVTGKGPLENLADHLA | 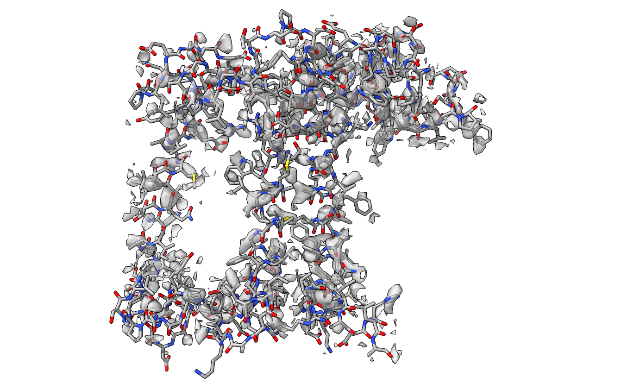 |
| R,r | **CP29** | LHCB4.2 | 28.16 | 5.63 | >Q07473  DRPLWYPGAISPDWLDGSLVGDRGFDPFGLGKPAEYLQFDIDSLDQNLAKNLAGDVIGTRTEAADAKSTPFQPYSEVFGIQRFRECELIHGRWAMLATLGALSVEWLTGVTWQDAGKVELVDGSSYLGQPLPFSISTLIWIEVLVIGYIEFQRNAELDSEKRLYPGGKFFDPLGLAADPEKTAQLQLAEIKHARLAMVAFLGFAVQAAATGKGPLNNWATHLSDPLHTTIIDTFS | 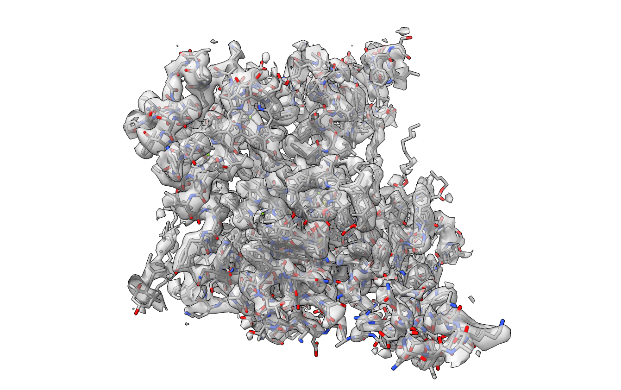 |
| S,s | **CP26** | LHCB5 | 25.21 | 4.97 | >Q9XF89  DELAKWYGPDRRIFLPDGLLDRSEIPEYLNGEVAGDYGYDPFGLGKKPENFAKYQAFELIHARWAMLGAAGFIIPEALNKYGANCGPEAVWFKTGALLLDGNTLNYFGKNIPINLVLAVVAEVVLLGGAEYYRITNGLDFEDKLHPGGPFDPLGLAKDPEQGALLKVKEIKNGRLAMFAMLGFFIQAYVTGEGPVENLAKHLSDPFGNNLLTVIAG | 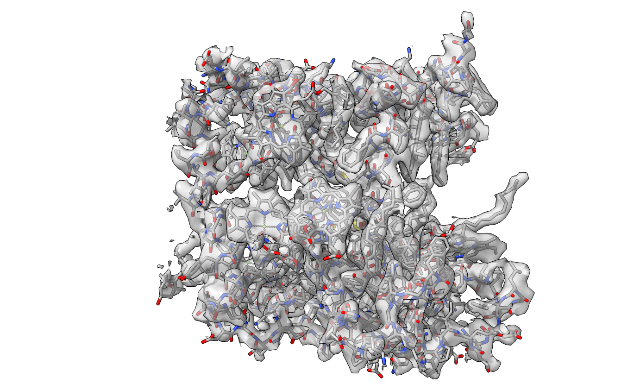 |
| 4,8 | **CP24** | LHCB6 | 23.11 | 5.10 | >Q9LMQ2  KKSWIPAVKGGGNFLDPEWLDGSLPGDFGFDPLGLGKDPAFLKWYREAELIHGRWAMAAVLGIFVGQAWSGVAWFEAGAQPDAIAPFSFGSLLGTQLLLMGWVESKRWVDFFNPDSQSVEWATPWSKTAENFANYTGDQGYPGGRFFDPLGLAGKNRDGVYEPDFEKLERLKLAEIKHSRLAMVAMLIFYFEAGQGKTPLGALG | 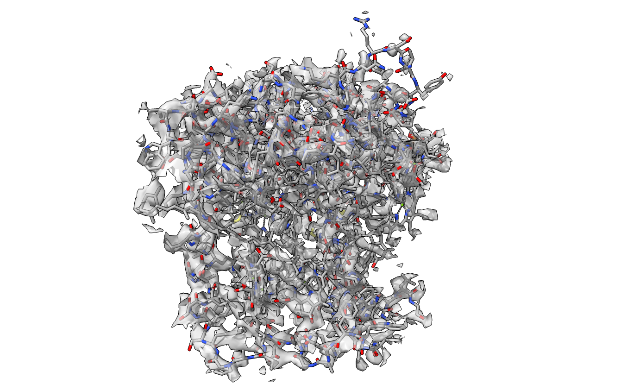 |

*Table S4 – Number and associated chains for the co-factors present in each higher plant PSII extracted at pH 7.5 (PDB: 7OUI, 5MDX, 5XNM, 3JCU). The chain IDs with associated ligands are highlighted in grey. Regarding the new Arabidopsis PSII model (7OUI), in the future certain associations of ligands to specific chains might differ, since PDB annotators might change this association if they will. To note that 3JCU is a C_2_S_2_ type of PSII complex while the remaining are C_2_S_2_M_2_ supercomplexes.*

|  | **Ligands** | | | | | |
| --- | --- | --- | --- | --- | --- | --- |
| **Ligand Name** | | **3-letter Code** | **7OUI** | **5MDX** | **5XNM** | **3JCU** |
| FE (II) ION | | FE2 | 2  (D, d) | 2  (A, a) | 2  (A, a) | 2  (A, a) |
| CHLOROPHYLL A | | CLA | 162  (2, 6, A, a, B, b, C, c, D, d, G, g, N, n, R, r, S, s, Y, y) | 212  (1, 2, 3, 4, 5, 6, 7, 8,A, B, C, D, G, N, R, S, Y, a, b, c, d, g, n, r, s, y) | 216  (1, 2, 3, 4, 5, 6, 7, 8,  A, B, C, D, G, N, R, S, Y, a, b, c, d, g, n, r, s, y) | 156  (A, B, C, D, G, N, R, S, Y, a, b, c, d, g, n, r, s, y) |
| PHEOPHYTIN A | | PHO | 4  (A, a) | 4  (A, D, a, d) | 4  (A, a) | 4  (A, a) |
| PROTOPORPHYRIN IX CONTAINING FE | | HEM | 2  (F, f) | 2  (E, e) | 2  (F, f) | 2  (F, f) |
| CHLOROPHYLL B | | CHL | 60  (1, 2, 5, 6, G, g, N, n, R, r, S, s, Y, y) | 96  (1, 2, 3, 4, 5, 6, 7, 8,G, N, Y, g, n, y) | 98  (1, 2, 3, 4, 5, 6, 7, 8,G, N, Y, g, n, y) | 50  (G, N, R, S, Y, g, n, r, s, y) |
| (3R,3'R,6S)-4,5-DIDEHYDRO-5,6-DIHYDRO-BETA,BETA-CAROTENE-3,3'-DIOL | | LUT | 18  (G, g, N, n, R, r, S, s, Y, y) | - | 32  (1, 2, 3, 4, 5, 6, 7, 8,G, N, R, S, Y, g, n, r, s, y) | 18  (G, N, R, S, Y, g, n, r, s, y) |
| (3S,5R,6S,3'S,5'R,6'S)-5,6,5',6'-DIEPOXY-5,6,5',6'- TETRAHYDRO-BETA,BETA-CAROTENE-3,3'-DIOL | | XAT | 2  (R, r) | - | 16  (1, 2, 3, 4, 5, 6, 7, 8,G, N, R, Y, g, n, r, y) | 8  (G, N, R, Y, g, n, r, y) |
| (3S,5R,6R,3'S,5'R,6'S)-5',6'-EPOXY-6,7-DIDEHYDRO- 5,6,5',6'-TETRAHYDRO-BETA,BETA-CAROTENE-3,5,3'-TRIOL; 9'-CIS-NEOXANTHIN | | NEX | 10  (G, g, N, n, R, r, S, s, Y, y) | - | 16  (1, 2, 3, 5, 6, 7,G, N, R, S, Y, g, n, r, s, y) | 10  (G, N, R, S, Y, g, n, r, s, y) |
| 1,2-DIPALMITOYL-PHOSPHATIDYL-GLYCEROLE | | LHG | 30  (2, 6, B, b, C, c, D, d, G, g, L, l, N, n, R, r, S, s, Y, y) | - | 36  (1, 2, 3, 4, 5, 6, 7, 8, B, C, D, G, L, N, R, S, Y,  b, c, d, g, l, n, r, s, y) | 18  (D, G, L, N, R, S, Y,  d, g, l, n, r, s, y) |
| BETA-CAROTENE | | BCR | 22  (A, a, B, b, C, c, D, d, H, h, T, t) | - | 24  (4, 8,A, B, C, D, H, T, a, b, c, d, h, t) | 20  (A, B, C, D, H, a, b, c, d, h) |
| CA-MN4-O5 CLUSTER | | OEX | - | - | 2  (A, a) | 2  (A, a) |
| 1,2-DI-O-ACYL-3-O-[6-DEOXY-6-SULFO-ALPHA-D-GLUCOPYRANOSYL]-SN-GLYCEROL | | SQD | 8  (A, a, B, b) | - | 8  (A, B, a, b) | 6  (A, B, a, b) |
| 1,2-DISTEAROYL-MONOGALACTOSYL-DIGLYCERIDE | | LMG | 12  (A, a, B, b, D, d, Z, z) | - | 14  (A, B, C, D, Z, a, b, c, d, z) | 10  (A, B, C, D, Z, a, b, c, d, z) |
| 2,3-DIMETHYL-5-(3,7,11,15,19,23,27,31,35-NONAMETHYL-2,6,10,14,18,22,26,30,34-HEXATRIACONTANONAENYL-2,5-CYCLOHEXADIENE-1,4-DIONE-2,3-DIMETHYL-5-SOLANESYL-1,4-BENZOQUINONE | | PL9 | 4  (A, a, D, d) | - | 4  (A, D, a, d) | 2  (D, d) |
| DIGALACTOSYL DIACYL GLYCEROL (DGDG) | | DGD | 8  (B, b, C, c, H, h) | - | 10  (B, C, H, b, c, h) | 8  (C, H, c, h) |
| BICARBONATE ION | | BCT | 2  (D, d) | - | 2  (D, d) | 2  (D, d) |
| CHLORIDE ION | | CL | 2  (D, d) | - | - | 4  (A, a) |
| DIGITONIN | | AJP | 22  (A, a, B, b, P, p) | - | - | - |
| CALCIUM ION | | CA | 4  (J, j, P, p) | - | - | - |
| WATER | | HOH | 139  (P, p, V, v) | - | - | - |

*Table S5 – The different PSII purification conditions and the effects on the OEC environment.*

|  | ***Arabidopsis thaliana*** | | ***Pisum sativum*** | | ***Spinacia oleracea*** |
| --- | --- | --- | --- | --- | --- |
|  | **7OUI** | **5MDX** | **5XNM** | **5XNL** | **3JCU** |
| **PsbP?** | NO | NO | NO | YES | YES |
| **PsbQ?** | NO | NO | NO | YES | YES |
| **Na^+^ as a ligand?** | NO | NO | YES | NO | YES |
| **Mn_4_CaO_5_?** | NO | NO | YES | YES | YES |
| **Starting Membranes** | BBY 1mg/mL washed with 10 mM HEPES-KOH pH [7.2 -7.5] | BBY 1mg/mL washed with 10 mM HEPES-KOH pH 7.5 | Thylakoid membranes 1 mg/mL,  in 5 mM MES pH 6.0, 10 mM NaCl, 5 mM MgCl_2_ and 2 M glycine betaine (MNMβ buffer) | | 500µg grana membranes (BBY?) washed with 1mM EDTA prior to solubilization |
| **Solubilization** | 0.5 mg/mL Chl  0.5% (w/v) Digitonin + 0.2% (w/v) Beta-DDM  30 minutes | 0.5 mg/mL Chl  0.5% (w/v) Digitonin +  0.2% (w/v) Alpha-DDM  30 minutes | 2% Alpha-DDM  10 mM HEPES pH 7.5 | 2.5% Alpha-DDM  10 mM HEPES pH 7.5 | 0.5 mg/mL Chl  0.3% Alpha-DDM  1 min vortexing  10 mM HEPES pH 7.5 |
| **Sucrose Gradient** | Freeze-thawing method.  0.35M sucrose gradient  10 mM HEPES-KOH pH 7.5  0.01% (w/v) digitonin | Freeze-thawing method.  0.65M sucrose gradient  10 mM HEPES-KOH pH 7.5  0.01% (w/v) digitonin | Freeze-thawing method  (-80 to 4°C).  0.65M sucrose gradient  10 mM HEPES pH 7.5 and 0.016% α-DDM | Freeze-thawing method  (-80 to 4°C).  0.65M sucrose gradient  25 mM MES pH 5.7,  5 mM CaCl_2_ and 0.03% α-DDM | Freeze-thawing method  (-80 to 4°C).  0.65M sucrose gradient  10 mM HEPES-KOH pH 7.5  0.008% α-DDM |
| **U centrifugation** | 13 000 g for 12 min.  41 000 rpm for 7h (200µg Chl) (SW41)  Last band was collected | 13 000 g for 10 min.  41 000 rpm for 17h (200µg Chl) (SW41) | 247 600 g, 4 °C for 20 h (SW41).  3^rd^ band collected | 100 000 g, 4 °C for 15 h (SW41).  3^rd^ band collected | 16 000 g for 10 min.  247 600g, 4 °C for 21 h (SW41).  B9 band Chl a/b ratio around 2.9–3.1 was collected. |
| **Final buffer and pH** | 10mM HEPES-KOH  pH 7.5 + 0.01% w/v digitonin | 10mM HEPES-KOH  pH 7.5 + 0.01% digitonin | 10mM HEPES  pH 7.5 | 25mM MES  pH 5.7 | 10mM HEPES  pH 7.5 |
| **Final Salt Concentration** | No salt (after BBY prep) | No salt (after BBY prep) | None | 5mM CaCl_2_ | None |
| **Concentration** | Concentrated to 3.5 mg/mL chl on on Millipore Amicon filter (30 kDa cut-off, 10mL) in rounds of 5.000 g for 30 min and washed in-between w/ 2mL 10mM HEPES-KOH pH 7.5 + 0.01% digitonin. Last concentration step with Vivaspin 500 (100kDa cut-off, 500µL). Final 20µL. | Concentrated to 3.5 mg/mL chl on Millipore Amicon filter (10 kDa cut-off, 10mL) in rounds of 3500 g for 20 min and washed in-between w/ 2mL 10mM HEPES-KOH pH 7.5 + 0.01% digitonin. Final 60µL. | 3 mg/mL (in chlorophyll) | | Concentrated to 3 mg/mL Chl by using a 100-kDa cut-off concentrator. |

*Table S6 – Inter-chlorophyll distances (Mg-Mg) of coupled pairs involved in energy transfer between PSII subunits.*

|  |  | | | | | | |  |  |  |  |  |
| --- | --- | --- | --- | --- | --- | --- | --- | --- | --- | --- | --- | --- |
| **Subunit and**  **Chlorophyll ID** | | **Subunit and**  **Chlorophyll ID** | **Mg-Mg**  **distance (Å)** | **Location** |  |  | **Subunit and**  **Chlorophyll ID** | | | **Subunit and**  **Chlorophyll ID** | **Mg-Mg**  **distance (Å)** | **Location** |
|  | | | | |  |  |  | | | | | |
| **M-LHCII_A_** | | **CP29** |  |  |  |  | **M-LHCII_A_** | | | **CP29** |  |  |
| CLA611 | | CLA611 | 16.4 | Stroma |  |  | CLA614 | | | CHL614 | 14.8 | Lumen |
|  | |  |  |  |  |  |  | | |  |  |  |
| **CP29** | | **CP47** |  |  |  |  | **S-LHCII_A_** | | | **CP29** |  |  |
| CLA603 | | CLA610 | 19.2 | Stroma |  |  | CHL605 | | | CHL606 | 18.4 | Lumen |
| CLA609 | | CLA616 | 21.8 | Stroma |  |  | CHL605 | | | CLA604 | 17.9 | Lumen |
| CLA613 | | CLA602 | 23.5 | Lumen |  |  |  | | |  |  |  |
|  | |  |  |  |  |  |  | | |  |  |  |
| **S-LHCII_A_** | | **CP43** |  |  |  |  |  | | |  |  |  |
| CLA611 | | CLA506 | 16.9 | Stroma |  |  |  | | |  |  |  |
| CLA612 | | CLA506 | 22.1 | Stroma |  |  |  | | |  |  |  |
| CLA614 | | CLA501 | 23.6 | Lumen |  |  |  | | |  |  |  |
|  | |  |  |  |  |  | **S-LHCII_B_** | | | **CP26** |  |  |
| **S-LHCII_B_** | | **CP26** |  |  |  |  | CHL605 | | | CLA604 | 19.2 | Lumen |
| CLA604 | | CLA604 | 24.4 | Lumen |  |  | CHL608 | | | CLA610 | 20.9 | Stroma |
| CLA610 | | CLA610 | 26.1 | Stroma |  |  | CHL608 | | | CLA612 | 21.0 | Stroma |
|  | |  |  |  |  |  |  | | |  |  |  |
| **CP26** | | **CP43** |  |  |  |  |  | | |  |  |  |
| CLA611 | | CLA513 | 16.7 | Stroma |  |  | **CP26** | | | **CP43** |  |  |
| CLA611 | | CLA512 | 17.6 | Stroma |  |  | CHL601 | | | CLA513 | 13.0 | Stroma |
| CLA614 | | CLA503 | 16.7 | Lumen |  |  |  | | |  |  |  |
